# Zebra optimization algorithm incorporating opposition-based learning and dynamic elite-pooling strategies and its applications

**DOI:** 10.1371/journal.pone.0329504

**Published:** 2025-08-05

**Authors:** Tengfei Ma, Guangda Lu, Zhuanping Qin, Tinghang Guo, Zheng Li, Changli Zhao

**Affiliations:** 1 School of Automation and Electrical Engineering, Tianjin University of Technology and Education, Tianjin, China; 2 Tianjin Key Laboratory of Information Sensing & Intelligent Control, Tianjin, China; 3 Tianjin Sino-German University of Applied Sciences, Tianjin, China; Yalova University, TÜRKIYE

## Abstract

To address the limitations of the Zebra Optimization Algorithm (ZOA), including insufficient late-stage optimization search capability, susceptibility to local optima, slow convergence, and inadequate exploration, this paper proposes an enhanced Zebra Optimization Algorithm integrating opposition-based learning and a dynamic elite-pooling strategy (OP-ZOA: Opposition-Based Learning Dynamic Elite-Pooling Zebra Optimization Algorithm). he proposed search algorithm employs a good point set-elite opposition-based learning mechanism to initialize the population, enhancing diversity and facilitating escape from local optima. Additionally, a real-time information synchronization mechanism is incorporated into the position update process, enabling the exchange of position and state information between the optimal individual (X_best_) and the vigilante agent (X_worse_). This eliminates information silos, thereby improving global search capability and convergence speed. Furthermore, a dynamic elite-pooling strategy is introduced, incorporating three distinct fitness factors. The optimal individual’s position is updated by randomly selecting from these factors, enhancing the algorithm’s ability to attain the global optimum and increasing its overall robustness. During experimental evaluation, the efficiency of OP-ZOA was verified using the CEC2017 test functions, demonstrating superior performance compared to seven recently proposed meta-heuristic algorithms (Bloodsucking Leech Algorithm (BSLO), Parrot Optimization Algorithm (PO), Polar Lights Algorithm (PLO), Red-tailed Hawk Optimization Algorithm (RTH), Bitterling Fish Optimization Algorithm (BFO), Spider Wasp Optimization Algorithm (SWO) and Zebra Optimization Algorithm (ZOA)). Finally, OP-ZOA exhibits distinct advantages in optimizing the APF (artificial potential field) method to address local optimum convergence issues. Specifically, it achieves faster iteration speeds across four different environments, with the planned path length after escaping local optima being shortened by an average of 7.55175 m (16.291%) compared to other optimization algorithms. These results confirm OP-ZOA’s enhanced optimization capability, significantly improving both escape efficiency from local optima and solution reliability.

## Introduction

In science and engineering design, numerous complex optimization problems exist, characterized by non-convexity, high nonlinearity, multi-peak behavior, and multi-variable interactions. Metaheuristic algorithms have emerged as a research hotspot for addressing such challenges in engineering applications due to their advantages of simple implementation, operational flexibility, and efficient optimization search capabilities [1]. These algorithms have been successfully applied to solve real-world problems, including neural network optimization [2], resource allocation [3], and target tracking [4].

Multi-objective complex optimization problems are prevalent in production and daily life, where conflicting objectives must be optimized simultaneously [5,6]. However, obtaining optimal solutions for such problems remains challenging. To address this, multi-objective evolutionary algorithms—capable of deriving multiple solutions in a single learning process—have been extensively studied and applied in recent years [7–9]. Among these, swarm intelligence optimization algorithms exhibit distinct advantages, including minimal parameter requirements, straightforward implementation, and independence from gradient information. These algorithms can efficiently identify optimal solutions for multi-objective problems under highly complex constraints and within reasonable computational time [10–12]. Notable examples include Particle Swarm Optimization (PSO) [13], Artificial Bee Colony (ABC) [14], Philoponella Prominens Optimizer (PPO) [15], Harris Hawk Optimization (HHO) [16], and Whale Optimization Algorithm (WOA) [17]. Evolutionary algorithms, typically inspired by biological evolution, offer strong global search capabilities and adaptability to high-dimensional, nonlinear problems. Representative examples include the Differential Evolution Algorithm (DE) [18] and the Love Evolution Algorithm (LEA) [19], among others.

In the context of ongoing technological advancements, significant attention has been devoted to the development and refinement of swarm intelligence algorithms across various applications, including positioning computation [20], traveler path planning [21], support vector machine optimization [22], robot pathfinding [23], power system control [24], and Internet of Things (IoT) routing protocol optimization [25,26]. For instance, the integration of the swarm intelligence Boids model with Deep Reinforcement Learning (DRL) enhances the efficiency of UAVs in pursuit-evasion tasks [27]. Similarly, a hybrid approach combining an Artificial Neural Network (ANN) and the Hybrid Cuckoo Search Algorithm (HCS) addresses complex fluid dynamics problems, solves Partial Differential Equations (PDEs), and analyzes convective heat transfer in straight-ribbed sheets with temperature-dependent thermal conductivity [28–30]. The Multi-Objective Particle Swarm Optimization (MOPSO) algorithm has been applied to solve multi-warehouse vehicle routing problems in refinery oil distribution [31], while the Cuckoo Search (CS) algorithm, combined with a dual-file strategy, optimizes classical truss structures [32]. Additionally, metaheuristic-based algorithms have been implemented to identify small fixed-wing unmanned aerial vehicle (UAV) systems [33]. These improved algorithms demonstrate high efficiency in locating optimal solutions for large-scale problems. Despite their advantages—such as strong adaptability in automatic search strategy adjustment, high robustness in handling complex engineering problems, suitability for distributed computation, and ease of implementation—some of these methods still face challenges related to premature convergence to local optima.

The Zebra Optimization Algorithm (ZOA) is a heuristic optimization algorithm inspired by the collective behavior of zebra groups in nature, proposed by Trojovská et al. [34] in 2022. It offers advantages such as a simple principle and ease of implementation, and since its introduction, numerous scholars have developed improvements to the algorithm. Although ZOA demonstrates certain optimization advantages compared to most metaheuristic algorithms, it still suffers from limitations such as low convergence accuracy and susceptibility to local optima. For instance, Hazem M. El-Hageen et al. [35] proposed the Chaotic Zebra Optimization Algorithm (CZOA), which integrates chaotic mapping with ZOA to enhance the lifetime of wireless sensor networks (WSNs). The chaotic mapping increases the algorithm’s randomness and search range, thereby improving search diversity and reducing the risk of premature convergence to local optima. Similarly, Mahmoud M. Elymany et al. [36] introduced a hybrid optimization approach combining ZOA with an artificial gorilla troop optimizer (GTO) strategy. This synergy enhances the maximum power point tracking (MPPT) process in photovoltaic (PV) and wind power systems, ensuring maximum energy output under varying weather conditions. Additionally, Sarada Mohapatra et al. [37] combined ZOA with an Adaptive Network-based Fuzzy Inference System (ANFIS), using ZOA to optimize ANFIS parameters for more accurate MPPT control in hybrid microgrids.

Despite the enhancement of the zebra algorithm’s optimization accuracy and speed by the aforementioned research, the following issues persist due to the incompleteness of the improvement method: (1) The initial population prior to the iterative updating of the algorithm exhibits a significant dependency on the initial conditions, resulting in inadequate robustness. (2) The strategy of interactively updating the individual populations is relatively single and mechanical, which cannot be targeted, and lacks the problem of a reasonable balance between global and local search. (3) The algorithm remains susceptible to local optimal traps, resulting in poor convergence accuracy. (4) The individual population position update method is not detailed enough. Based on the above issues, the primary contributions of this study can be outlined as follows:

In this study, we propose a new zebra optimization algorithm (OP-ZOA, Opposition-Based Learning Dynamic Elite-Pooling Zebra Optimization Algorithm) that integrates opposition-based learning and dynamic elite-pooling strategies. The proposed approach implements three key innovations: First, a good point set-elite opposition-based learning mechanism initializes the population to enhance diversity. Second, a real-time information synchronization mechanism updates searcher positions by coordinating optimal individuals and vigilantes, enabling more effective defense strategy adaptation and risk management while boosting global search capability and convergence speed. Third, a dynamic elite-pooling strategy introduces three distinct fitness factors (mean fitness, sub-fitness, and elite fitness) for optimal individual position updates through randomized selection, significantly improving global optimization accuracy and solution quality. The algorithm’s superiority is rigorously validated through comprehensive benchmark testing on standard functions. Comparative analysis of engineering applications demonstrates OP-ZOA’s superior feasibility and performance relative to existing optimization methods.

### Traditional Zebra Optimization Algorithm

A **Initialization process**

The starting position of the zebra within the search area was chosen randomly. The ZOA population matrix XTheexperimentalenvironmentemployedinthis is shown in Eq 1.


$$X=[\begin{array}{ccc} x_{11} & x_{12} & \begin{array}{ccc} x_{13} & \cdots & x_{1d} \end{array}\\ x_{21} & x_{22} & \begin{array}{ccc} x_{23} & \cdots & x_{2d} \end{array}\\ \begin{array}{c} \vdots \\ x_{n1} \end{array} & \begin{array}{c} \vdots \\ x_{n2} \end{array} & \begin{array}{ccc} \begin{array}{c} x_{ij}\\ x_{n3} \end{array} & \begin{array}{c} \vdots \\ \cdots \end{array} & \begin{array}{c} \vdots \\ x_{nd} \end{array} \end{array} \end{array}]$$ (1)

where X is an n×d population matrix, d is the problem dimension, and n is the population size. $X_{i}$ is a vector representing a solution, and $x_{ij}$ is a variable in the solution.

ZOA members are updated using two natural behaviors of zebras. The first of these two behavioral patterns is foraging and the second is a predator defense mechanism. As a result, individuals in the ZOA community are renewed twice in each iteration.

The Zebra Optimization Algorithm (ZOA) [34] performs an optimization search by simulating the behavior of zebras to achieve position updating and solve the problem to be optimized, which is divided into two main phases: foraging strategy and defense strategy.

B **Foraging strategy**

The foraging strategy of ZOA is mainly modeling the role of the pioneer zebra [38,39], i.e., the best member of the population is regarded as the pioneer zebra, and the pioneer zebra leads the other members of the population towards its position in the search space so that all the members find their own positions.

Therefore, zebra’s position update during the foraging phase can be mathematically modeled using (2) and (3).


$$x_{i,j}^{new,P1}=x_{i,j}+r*\left(PZ_{j}-I*x_{i,j}\right)$$ (2)

$$X_{i}=\left\{ \;\;\;\begin{array}{c} \begin{array}{cc} X_{i}^{new,P1}, & F_{i}^{new,P1}<F_{i} \end{array}\\ \begin{array}{cc} X_{i,} & else \end{array} \end{array}\right.\;$$ (3)

where $X_{i}^{new,P1}$ is the new state of the ith zebra, $x_{i,j}^{new,P1}$is its jth dimension value, $F_{i}^{new,P1}$ is the value of its objective function, PZ is the pioneering zebra that is the best member, $PZ_{j}\;$is its jth dimension, r is a random number in the interval [0,1], and I=round(1+rand), where rand is a random number in the interval [0,1]. Thus, I∈{1,2}, and if the parameter I=2, the variation in population mobility is much larger.

C **Defense strategy**

The defense strategy of ZOA mainly simulates the defense strategy of zebras against predator attacks [40,41], thus updating the position of ZOA group members in the search space. The defense strategy consists of two scenarios: (2) escape and (3) launching an attack. The escape strategy mainly simulates the zebras using zigzag escape routes and random side-turns, which can be mathematically modeled by S1 in Eq 4; the launching an attack strategy mainly simulates that when a certain zebra is attacked, the other zebras will move towards the attacked zebra and try to scare and confuse the attacker by establishing a defensive structure. Its mathematical modeling can be simulated by S2 in Eq 4. When updating the zebra’s position, the new position is accepted if the zebra has a higher objective function value at the new position. This updating condition can be modeled by Eq 5.


$$x_{i,j}^{new,P2}=\left\{ \;\;\;\begin{array}{c} \begin{array}{cc} S1\;\;:x_{i,j}+R*(2r-1)*\left(1-\frac{t}{T}\right)*x_{i,j}, & P_{s}\leq 0.5 \end{array}\\ \begin{array}{cc} S2:x_{i,j}+r*\left(AZ_{j}-I*x_{i,j}\right) & else \end{array} \end{array}\right.\;$$ (4)

$$X_{i}=\left\{ \;\;\;\begin{array}{c} \begin{array}{cc} X_{i}^{new,P2}, & F_{i}^{new,P2}<F_{i} \end{array}\\ \begin{array}{cc} X_{i}, & else \end{array} \end{array}\right.\;$$ (5)

where $X_{i}^{new,P2}$ is the new state of the ith zebra based on the defense strategy, $x_{i,j}^{new,P2}$ is its jth dimension value, $F_{i}^{new,P2}$ is the value of its objective function, t is the iterative contour, T is the maximum number of iterations, R is a constant equal to 0.01, $R_{s}$ is the randomly generated in the interval [0,1] of two strategies probability of choosing one, AZ is the state of the attacked zebra, and $AZ_{j}$ is its jth dimension value.

Each ZOA iteration updates the population members based on the foraging and defense strategies. The process of updating the algorithm population continues according to (2) to (5) until the algorithm is fully executed. During successive iterations, the best candidate solution is updated and saved.

### Improved Zebra Optimization Algorithm

This section presents an enhanced Zebra Optimization Algorithm (OP-ZOA, Opposition-Based Learning Dynamic Elite-Pooling Zebra Optimization Algorithm) that combines opposition-based learning with a dynamic elite-pooling strategy. The proposed hybrid algorithm introduces three key modifications to the classical ZOA framework: (1) population initialization via good point set-elite opposition-based learning, (2) incorporation of a real-time information synchronization mechanism, and (3) implementation of a dynamic elite-pooling strategy. These enhancements collectively improve the algorithm’s global search capability and convergence speed while significantly strengthening its ability to escape local optima.

A **Good point set-elite opposition-based learning-initializing populations.**
   a **Good point set initialization populations**

The good point set initialization population generates more uniformly distributed population nodes compared to traditional random initialization. This approach significantly reduces the impact of randomness, yielding more stable and reliable results across multiple algorithm runs while improving solution space coverage. By addressing the inherent limitations of pseudorandom number generation systems – particularly their tendency to produce non-uniform population distributions and clustering effects – this initialization strategy enhances the algorithm’s ability to achieve global optimal solutions.

Let $G_{s}$ be a unit cube in s-dimensional space, and if $r\in G_{s}$, where there exists a set of points:


$$\;P_{n}(k)=\{(\;\;\;\begin{array}{c} \left\{r_{1}^{\left(n\right)}*k\right\},\cdots ,\left\{r_{s}^{\left(n\right)}*k\right\} \end{array}\;\;\;\;\;\;),1\leq k\leq n\}$$ (6)

Its deviation satisfies >$\;\varphi (\;\;\;\begin{array}{c} n \end{array}\;\;\;\;\;\;)=C\left(\;\;\;\begin{array}{c} r,\varepsilon \end{array}\;\;\right)n^{-1+\varepsilon }$, where  C(r,ε) is a constant related only to r and ε(ε>0), then $P_{n}(k)$ is called the good point set.

The value of the good point set r is r={2cos(2πk/p),1≤k≤s}, where p is the smallest prime number satisfying (p−3text/2≥s. After generating the good points set, it is mapped to the search space:


$$X_{i,j}=(ub-lb)*r+lb$$ (7)

where ub is the upper bound and lb is the lower bound.

**Fig 1:**
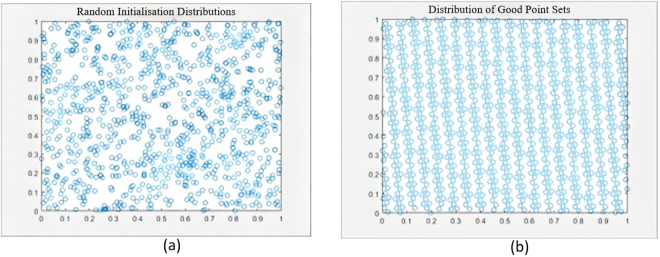
(a) Random initialized population distribution (b) Good point set initialized population distribution.

Fig 1 compares the population distributions between random initialization and good point set initialization. The good point set method demonstrates superior spatial uniformity, which enhances population diversity during the search process and effectively prevents premature convergence. This uniform distribution characteristic enables the algorithm to approach global optima more efficiently, thereby significantly improving optimization performance.

b **Elite opposition-based learning strategy**

After population initialization, randomly generated solutions often cause the algorithm to revisit undesired regions of the search space, leading to inefficient search.

To address this limitation, we implement an Opposition-Based Learning (OBL) strategy to enhance initial population diversity. Originally proposed by Tizhoosh [42] in 2005, OBL operates as a perturbation mechanism that generates inverse solutions ($\tilde{X}$) for each candidate solution (X) during initialization. The method evaluates both original and opposition solutions, retaining the superior candidates to improve solution quality.

The opposition-based learning approach generates inverse solutions ($\tilde{X}$) for each candidate solution (X) during the search process. This strategy significantly increases the probability of locating global optima compared to purely random sampling, as the opposite solutions provide complementary exploration of the search space. By simultaneously evaluating both original and opposition solutions, the algorithm enhances its detection capability while achieving more comprehensive search space coverage.

Suppose X∈R,X∈[a,b]. The opposite of X, $\tilde{X}$ is computed as follows:


$$\tilde{X}=a+b-X$$ (8)

The equation is generalized to the multidimensional case. $X_{i}\in R,X_{i}\in \left[a_{i},b_{i}\right]$. When defined, the equation is as follows:


$$\tilde{X_{i}}=a_{i}+b_{i}-X_{i,}i=1,2,3,\ldots .,n$$ (9)

The Opposition-Based Learning mechanism is added to the traditional ZOA algorithm, where ub and lb are the upper and lower bounds of the problem and X is the search agent, and the equation is as follows:


$$\tilde{X}=r*(ub-lb)-X$$ (10)

Individual X is selected based on the magnitude of the fitness of the greedy mechanism by comparing the fitness of X with $\tilde{X}$:


$$X=\left\{ \;\;\;\begin{array}{c} X,fit(X)<fit\left(\tilde{X}\right)\\ \tilde{X},fit\left(X^{\prime }\right)<fit(X) \end{array}\right.\;$$ (11)

B **Mechanisms for synchronizing real-time information**

The traditional ZOA exhibits limited information exchange between individuals during iterations. When encountering potential threats or “invaders”, the population frequently fails to coordinate effective collective defense mechanisms. This communication deficiency significantly constrains the algorithm’s capacity for rapid environmental adaptation. The traditional ZOA exhibits limited information exchange between individuals during iterations. When encountering potential threats or invaders, the population frequently fails to coordinate effective collective defense mechanisms. This communication deficiency significantly constrains the algorithm’s capacity for rapid environmental adaptation.

To address these limitations, this study introduces a real-time information synchronization mechanism to improve population responsiveness and environmental adaptation. The mechanism comprises two key components: the optimal individual (X_best) and the vigilante agent (X_worse). The optimal individual X_best guides collective foraging behavior while continuously updating and broadcasting its position through real-time synchronization, enabling efficient exploitation of resource-rich regions. Simultaneously, the vigilante X_worse, representing the population’s least adapted member, monitors potential threats and approaching intruders during iterations. Through synchronized position and status updates from both components, the population achieves more effective defense strategy adjustments and risk mitigation.

The population renewal method is shown in Eq 12:


$$X=X+\beta_{1}*\left(X_{best}-X\right)+\beta_{2}*\left(X-X_{worse}\right)$$ (12)

where $\beta_{1}$ is the bootstrap factor and$\;\beta_{2}$ is the caution factor with a random number of size [0,1] respectively.

Through the mechanism of Real-time information synchronization, ZOA is able to respond more flexibly to complex and changing environmental conditions and enhance the overall survivability of the population, while achieving a more efficient exploration and utilization of equilibrium in the optimization problem solving process.

C **Dynamic elite-pooling strategy**

The Traditional ZOA often suffers from premature convergence to local optima during optimization, significantly limiting its exploration capability for potentially superior solutions. To address this limitation, we introduce a dynamic elite-pooling strategy designed to enhance global search performance and maintain population diversity. The strategy establishes an elite pool (Elitep) comprising the highest-fitness individuals, effectively preventing local optima entrapment.

The implementation involves three key steps: First, fitness evaluation identifies three optimal zebra individuals (PZ_1_, PZ_2_, PZ_3_). Second, their positional arithmetic mean ($PZ_{m}$) is computed as:


$$PZ_{m}=\frac{\left(PZ1+PZ2+PZ3\right)}{3}$$ (13)

The elite pool incorporates these three individuals along with their average position ($PZ_{m}$). During each iteration, rather than exclusively using the optimal individual’s position for updates, the algorithm randomly selects a reference point from Elitep to guide zebra movement. This dynamic elite-pooling approach achieves dual objectives: maintaining awareness of the current optimal solution while simultaneously exploring potential solution spaces suggested by other high-fitness individuals. The strategy significantly improves the algorithm’s exploration capability and local optima avoidance.

**Fig 2:**
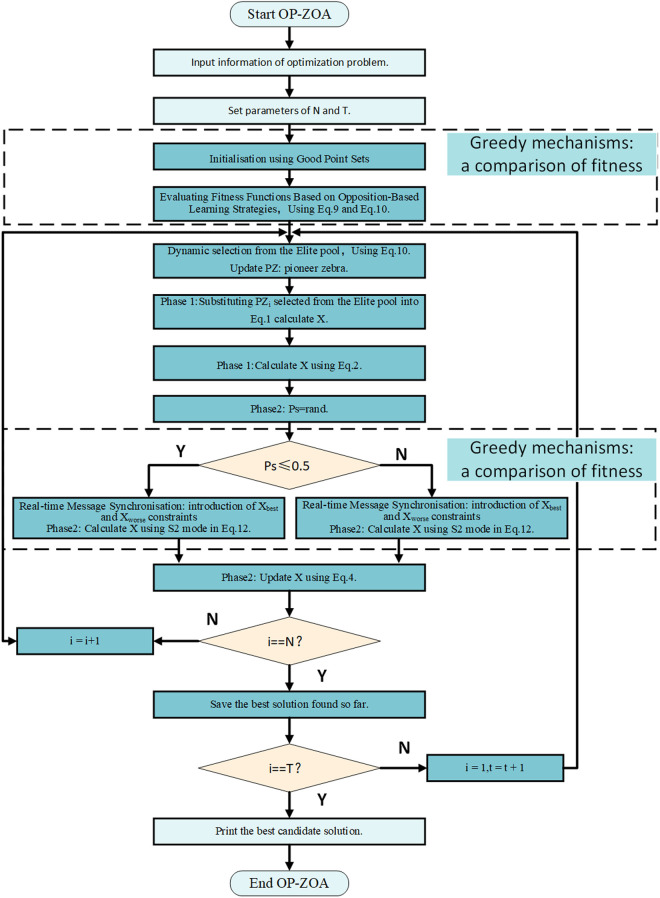
Flowchart of OP-ZOA algorithm.

The proposed OP-ZOA algorithm whose pseudo-code is shown in Algorithm 1, and the main process is shown in the form of a flowchart showing the various steps of OP-ZOA, as shown in Fig 2.

Algorithm 1: Pseudo-Code of OP-ZOA

Start OP-ZOA.

1. Input: The optimization problem information.

2. Set the number of iterations (T) and the number of zebras’ population (N).

3. Initialization of the position of zebras and evaluating Fitness Functions Based on Opposition-Based Learning Strategies, Using Eqs 9 and 10.

4. For t = 1: T

5.    Dynamic selection from the Elite pool, Using Eq 10 update PZ_i_: pioneer zebra.

6.    For i = 1: N

7.     **Phase 1: Foraging behavior**

8.     Substituting PZ_i_ selected from the Elite pool into Eq 1 new status of the ith zebra.

9.     Update the ith zebra using Eq. 2.

10.     **Phase 2: Defense strategies against predators**

11.     If Ps < 0.5, Ps = rand

12.       Strategy 1: against lion (exploitation phase)

13.       Real-time Message Synchronization: introduction of X_best_ and X_worse_ constraints: calculate new status of the ith zebra using mode S1 in Eq 12.

14.     else

15.       Strategy 2: against other predator (exploration phase)

16.       Calculate new status of the ith zebra using mode S2 in Eq. 12.

17.     end if

18.     Update the ith zebra using Eq 4.

19.   end for i = 1: N

20.   Save best candidate solution so far.

21. end for t = 1: T

22. Output: The best solution obtained by ZOA for given optimization problem.

End OP-ZOA

D **Analysis of the computational complexity**

The complexity of any algorithm is a function that provides the running time or space concerning the input size. This is of two kinds: one is the complexity of space and the other is time complexity. The OP-ZOA mainly consists of three parts, which are the initialization stage, foraging behavior stage and defense strategy stage. Thus, the computational complexity of the OP-ZOA algorithm mainly depends on these three stages. The time complexity of the initialization phase is O(N×D). The iterative computation consists of two phases: foraging behavior with a time complexity of O(N×D)+O(N) and defense strategy with identical complexity. Therefore, the algorithm’s total time complexity amounts to O(T×N×D). In terms of space complexity, the complexity of the population matrix X is O(N×D), the complexity of the fitness array fit is O(N), and the complexity of the fitness curve is O(T). Therefore, the total space complexity is O(N×D+T). Here, N represents the population size, D represents the problem dimension, and T represents the maximum number of iterations.

### Ablation experiments

A **CEC2017 functions**

The CEC2017 test functions are 30 classical test functions, and their benchmark functions are shown in Table 1. Functions F1-F3 represent unimodal functions featuring a single global optimum, designed to evaluate an algorithm’s capability for single-solution optimization. The multimodal functions F4-F9 contain multiple local optima alongside one global optimum, testing an algorithm’s ability to avoid premature convergence. Hybrid functions F11-F20 combine unimodal and multimodal characteristics to assess performance on mixed-type optimization problems. Composition functions F21-F29 incorporate complex nonlinear and nonconvex features, challenging algorithms with highly complex optimization scenarios. Notably, functions F11-F29 consist of mixed and combined compositions of benchmark functions, providing comprehensive evaluation metrics for algorithm testing.

**Table 1:** CEC2017 basic functions.

Function
F1Bent Cigar Function $\begin{array}{c} f_{1}(x)=x_{1}^{2}+10^{6}\sum_{i=2}^{D}x_{i}^{2} \end{array}$
F2Sum of Different Power Function $\begin{array}{c} f_{2}(x)=\sum_{i=1}^{D}{\left|x_{i}\right|}^{i+1} \end{array}$
F3Zakharov Function $\begin{array}{c} f_{3}(x)=\sum_{i=2}^{D}x_{i}^{2}+{\left(\sum_{i=1}^{D}0.5x_{i}\right)}^{2}+{\left(\sum_{i=1}^{D}0.5x_{i}\right)}^{4} \end{array}$
$F4Rosenbrock^{\prime }s\;Function$ $\begin{array}{c} f_{4}(x)=\sum_{i=1}^{D-1}\left(100{\left(x_{i}^{2}-x_{i+1}\right)}^{2}+{\left(x_{i}-1\right)}^{2}\right) \end{array}$
$F5Rastrigin^{\prime }s\;Function$ $\begin{array}{c} f_{5}(x)=\sum_{i=1}^{D}\left(x_{i}^{2}-10\cos(2\pi x_{i})+10\right) \end{array}$
$F6Expanded\;Schaffer^{\prime }s\;F6\;Function$ $Schaffer^{\prime }s\;F6\;Function:g(x,y)=0.5+\frac{\left({\text{sin}}^{2}\left(\sqrt{x^{2}+y^{2}}\right)-0.5\right)}{{\left(1+0.001(x^{2}+y^{2})\right)}^{2}}$ $f_{6}(x)=g\left(x_{1},x_{2}\right)+g\left(x_{2},x_{3}\right)+\ldots +g\left(x_{D-1},x_{D}\right)+g\left(x_{D},x_{1}\right)$
F7Lunacek bi−Rastrigin Funtion $\begin{array}{c} f_{7}(x)=\min\left(\sum_{i=1}^{D}\hat{{(x}_{i}}-\mu_{0}{)}^{2},dD+s\sum_{i=1}^{D}\hat{{(x}_{i}}-\mu_{1}{)}^{2}+10\left(D-\sum_{i=1}^{D}cos(2\pi \hat{z_{i})}\right)\right) \end{array}$ $\mu_{0}=2.5,\mu_{1}=-\sqrt{\frac{\mu_{0}-d}{s}},s=1-\frac{1}{2\sqrt{D+20}-8.2},d=1$ $y=\frac{10(x-o)}{100},\hat{x_{i}}=2sign\left({x_{i}}^{*}\right)y_{i}+\mu_{0},for\;i=1,2,\ldots ,D$ $z=\wedge^{100}(\hat{x}-\mu_{0})$
$F8Non-continuous\;Rotated\;Rastrigin^{\prime }s\;Function$ $\begin{array}{c} f_{8}(x)=\sum_{i=1}^{D}\left(z_{i}^{2}-10cos\left(2\pi z_{i}\right)+10\right)+f_{13}^{*} \end{array}$ $\begin{array}{cc} & \hat{x}={𝐌}_{1}\;\frac{5.12(x-o)}{100},y_{i}=\left\{ \begin{array}{cc} {\hat{x}}_{i} & if\left|{\hat{x}}_{i}\right|\leq 0.5\\ round\left(2{\hat{x}}_{i}\right)/2 & if\left|{\hat{x}}_{i}\right|>0.5 \end{array}\right.\;,\;for\;i=1,2,...,D\\ & z={𝐌}_{1}\Lambda^{10}{𝐌}_{2}T_{axy}^{0.2}\left(T_{oxz}(y)\right) \end{array}$ Where ${𝛬}^{\alpha }$:a diagonal matrix in D dimensions with the $\iota^{th}$ diagonal element as $\lambda_{ii}=\alpha^{\frac{i-1}{2(D-1)}}$, i=1,2,…,D. $T_{asv}^{\beta }:$if $x_{i}>0,x_{i}=x_{i}^{1+\beta \frac{i-1}{D-1}\sqrt{x_{i}}}$,for $i=1,\ldots ,D^{\left[4\right]}$ $T_{osz\;}:for$ $\begin{array}{c} x_{i}=sign\left(x_{i}\right)\exp\left({\hat{x}}_{i}+0.049\left(\sin\left(c_{1}{\hat{x}}_{i}\right)+\sin\left(c_{2}{\hat{x}}_{i}\right)\right)\right) \end{array}$, for i=1and $D^{\left[4\right]}$ $where\;{\hat{x}}_{i}=\left\{ \begin{array}{cc} \log\left(\left|x_{i}\right|\right) & \;if\;x_{i}\neq 0\\ 0 & \text{ otherwise} \end{array}\right.\;,sign\left(x_{i}\right)=\left\{ \begin{array}{cc} -1 & if\;x_{i}<0\\ 0 & if\;x_{i}=0\\ 1 & \text{otherwise} \end{array}\right.\;$ $c_{1}=\left\{ \begin{array}{cc} 10 & \text{if }x_{i}>0\\ 5.5 & \text{otherwise} \end{array}\right.\;,\text{and }c_{2}=\left\{ \begin{array}{cc} 7.9 & \text{if }x_{i}>0\\ 3.1 & \text{otherwise} \end{array}\right.\;$
F9Levy Function $\begin{array}{c} f_{9}(x)=\sin\left(\pi \omega_{1}\right)+\sum_{i=1}^{D-1}{\left(w_{i}-1\right)}^{2}\left[1+10\sin^{2}\left(\pi \omega_{i}+1\right)\right]+\left(w_{D}+1\right)\left[1+\sin^{2}\left(2\pi \omega_{D}\right)\right] \end{array}$ $Where\;\omega_{i}=1+\frac{x_{i-1}}{4},\forall i=1,\ldots D$ Continue
$F10Modified\;Schwefel^{\prime }s\;Function$ $\begin{array}{cc} & f_{10}(x)=418.9829\times D-\sum_{i=1}\;g(z_{i}),\;z_{i}=x_{i}+4.209687462275036e+002 \end{array}$ $\begin{array}{c} g(z_{i})=\left\{ \;\;\;\;\;\;\begin{array}{c} z_{i}\sin\left({\left|z_{i}\right|}^{\frac{1}{2}}\right)\text{if }\left|z_{i}\right|\leq 500\\ \left(500-\text{mod}\left(z_{i},500\right)\right)\sin\left(\sqrt{\left|500-\text{mod}\left(z_{i},500\right)\right|}\right)-\frac{(z_{i}-500{)}^{2}}{10000D}\text{if}{\;z}_{i}>500\\ \;\left(\text{mod}\left(\left|z_{i}\right|,500\right)-500\right)\sin\left(\sqrt{\left|\text{mod}\left(\left|z_{i}\right|,500\right)-500\right|}\right)-\frac{(z_{i}+500{)}^{2}}{10000D}\text{if }z_{i}<-500 \end{array}\right. \end{array}$
F11High Conditioned Elliptic Function $\begin{array}{c} f_{11}(x)=\sum_{i=1}^{D}{\left(10^{6}\right)}^{\frac{i-1}{D-1}}x_{i}^{2} \end{array}$
F12Discus Function $\begin{array}{c} f_{12}(x)=10^{6}x_{1}^{2}+\sum_{i=2}^{D}x_{i}^{2} \end{array}$
$F13Ackley^{\prime }s\;Function$ $\begin{array}{c} f_{13}(x)=-20exp(-0.2\sqrt{\frac{1}{D}\sum_{i=1}^{D}\;x_{i}^{2}})-exp(\frac{1}{D}\sum_{i=1}^{D}\;cos(2\pi x_{i}))+20+\text{e} \end{array}$
F14Weierstrass Function $\begin{array}{c} f_{14}(x)=\sum_{i=1}^{D}\;\left(\sum_{k=0}^{k\max}\;[a^{k}cos(2\pi b^{k}(x_{i}+0.5))]\right)-D\sum_{k=0}^{k\max}\;[a^{k}cos(2\pi b^{k}\cdot 0.5)]\;a=0.5,b=3,kmax=20 \end{array}$
$F15Griewank^{\prime }s\;Function$ $\begin{array}{c} f_{15}(x)=\sum_{i=1}^{D}\;\frac{x_{i}^{2}}{4000}-\prod_{i=1}^{D}\;cos(\frac{x_{i}}{\sqrt{i}})+1 \end{array}$
F16Katsuura Function $\begin{array}{c} f_{16}(x)=\frac{10}{D^{2}}\prod_{i=1}^{D}\;{\left(1+i\sum_{j=1}^{32}\;\frac{\left|2^{j}x_{i}-round(2^{j}x_{i})\right|}{2^{j}}\right)}^{\frac{10}{D^{12}}}-\frac{10}{D^{2}} \end{array}$
F17HappyCat Function $\begin{array}{c} f_{17}(x)={\left|\sum_{i=1}^{D}\;x_{i}^{2}-D\right|}^{1/4}+\left(0.5\sum_{i=1}^{D}\;x_{i}^{2}+\sum_{i=1}^{D}\;x_{i}\right)/D+0.5 \end{array}$
F18HGBat Function $\begin{array}{c} f_{18}(x)={\left|{\left(\sum_{i=1}^{D}\;x_{i}^{2}\right)}^{2}-{\left(\sum_{i=1}^{D}\;x_{i}\right)}^{2}\right|}^{1/2}+\left(0.5\sum_{i=1}^{D}\;x_{i}^{2}+\sum_{i=1}^{D}\;x_{i}\right)/D+0.5 \end{array}$
$F19EXpanded\;Griewank^{\prime }s\;plus\;Rosenbrock^{\prime }s\;Function$ $f_{19}(x)=f_{7}(f_{4}(x_{1},x_{2}))+f_{7}(f_{4}(x_{2},x_{3}))+...+f_{7}(f_{4}(x_{D-1},x_{D}))+f_{7}(f_{4}(x_{D},x_{1}))$
$F20Schaffer^{\prime }s\;Function$ $\begin{array}{c} f_{20}(x)={\left[\frac{1}{D-1}\sum_{i=1}^{D-1}\;\left(\sqrt{s_{i}}.(sin(50.0s_{i}^{0.2})+1)\right)\right]}^{2},s_{i}=\sqrt{x_{i}^{2}+x_{i+1}^{2}} \end{array}$

B **Experimental design**

The experimental environment employed in this study is Windows 11 64-bit operating system, with an Intel® Core™ i9-14900HXHz CPU operating at a main frequency of 2.20GHz. The test software utilized is MATLAB R2022a.

This study introduces three key enhancements to the traditional zebra optimization algorithm (ZOA): (1) good point set-elite opposition-based learning for population initialization, (2) a dynamic elite-pooling strategy, and (3) a real-time information synchronization mechanism. Each enhancement was systematically integrated into ZOA and evaluated independently through controlled experiments to assess its individual contribution to algorithm performance.

**Fig 3:**
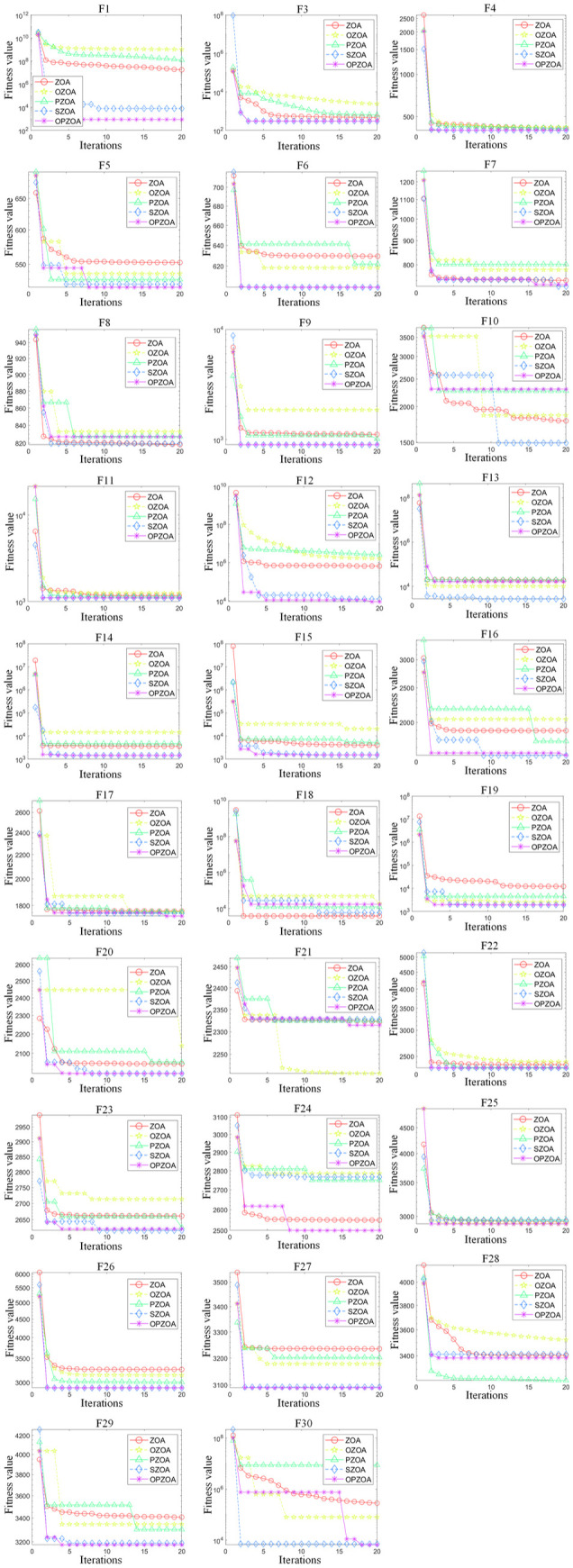
Iterative curve of ablation experiment.

**Fig 4:**
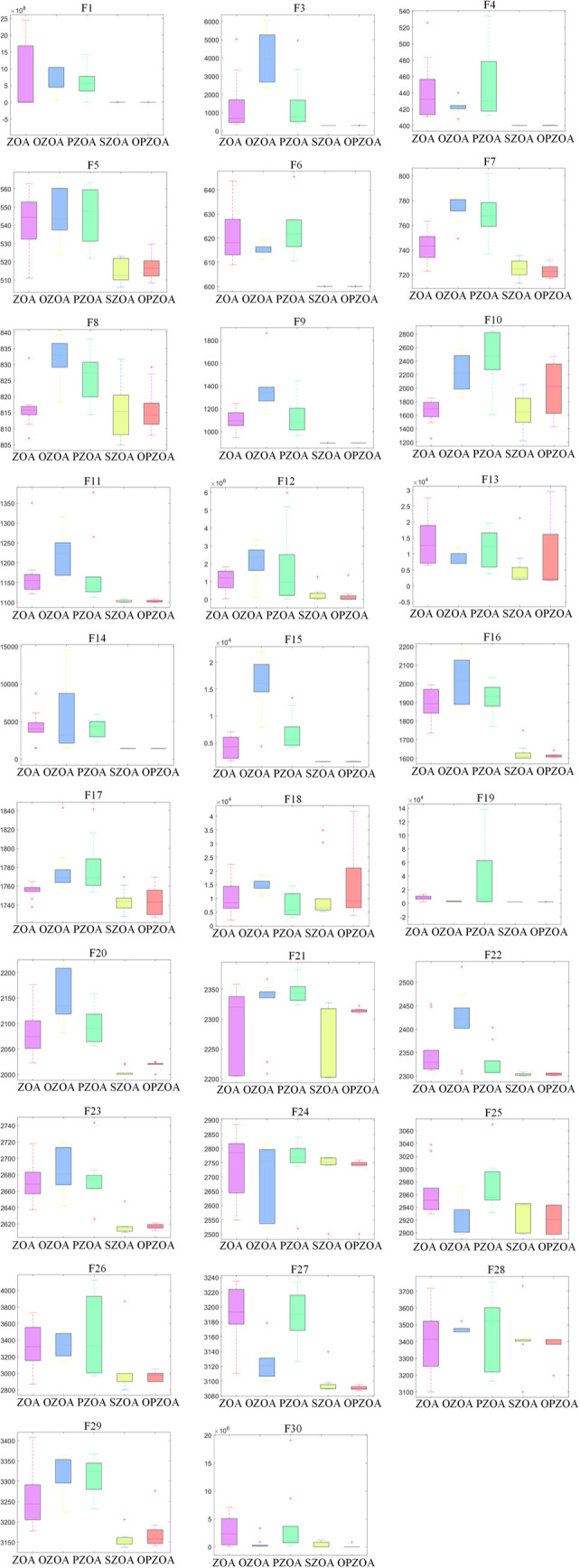
Box plot of ablation experiment data.

To ensure fair comparison, all five algorithms were configured with identical parameters: search intervals between [−100,100], population size of 30, 10-dimensional search space, and maximum iteration count of 500. Performance evaluation employed comprehensive statistical measures including minimum (Min), mean (Mean), standard deviation (Std), maximum (Max), Wilcoxon rank-sum test, Wilcoxon significance test, and Friedman test, with detailed results presented in Tables 2 and 3 and Figs 3 and 4. Specifically, the OZOA algorithm integrates improvement point 1, the PZOA algorithm integrates improvement point 2, and the SZOA algorithm integrates improvement point 3. The serial numbers 1, 2, 3, 4 and 5 in the table correspond to ZOA and OZOA, respectively, PZOA, SZOA, and OP-ZOA algorithms.

**Table 2:** Ablation comparison experimental data.

F1	Min	Max	Mea	Std	Friedman rank
1	181066.3701	2451641978	691959338.9	1034628034	3.6
2	63276915.94	1093264952	642888254.5	360346972.7	4.1
3	297444.7946	1419456870	587705240.4	414301048.4	4.3
4	2652.61656	53839.47241	11687.82467	15192.37839	1.7
**5**	**134.3877904**	**11839.84962**	**4070.715284**	**4353.617822**	**1.3**
F3	Min	Max	Mea	Std	Friedman rank
1	354.1835963	5034.771925	1398.151347	1568.353324	3.7
2	2371.122014	6073.033982	4066.920389	1537.341869	4.8
3	408.5645595	4960.571278	1444.233291	1528.486804	3.5
4	300	300	300	3.28186E-14	1.55
**5**	**300**	**300**	**300**	**2.67962E-14**	**1.45**
F4	Min	Max	Mea	Std	Friedman rank
1	410.4980672	525.696363	443.3039008	37.12938224	4
2	408.0339295	439.9866464	423.2333365	8.182469903	3.8
3	412.3016739	533.8851747	448.001191	42.85023617	4.2
4	400.0598474	400.1976045	400.1248734	0.048499371	1.3
**5**	**400.0999088**	**400.3874161**	**400.2225177**	**0.098545007**	**1.7**
F5	Min	Max	Mea	Std	Friedman rank
1	511.0638219	562.7616062	542.0716396	15.85678747	3.6
2	523.8936877	566.2705127	547.0278321	13.35258974	4
3	521.7827564	563.4770853	544.8046898	14.95964033	4
4	506.0494921	523.0313837	514.7591145	6.192498851	1.6
**5**	**508.4601938**	**529.6498949**	**517.5200171**	**6.879303024**	**1.8**
F6	Min	Max	Mea	Std	Friedman rank
1	608.9219843	643.6326681	621.2213002	10.66490157	4
2	611.2879801	619.2335692	615.2710326	2.422795082	3.5
3	610.5084256	645.4534469	622.7438238	9.729185598	4.5
4	600	600.0000485	600.0000054	1.51944E-05	1.25
**5**	**600**	**600.0000572**	**600.0000177**	**1.75494E-05**	**1.75**
F7	Min	Max	Mea	Std	Friedman rank
1	722.8939458	763.3936213	742.5796422	12.11951493	3.2
2	749.3111541	785.5886643	773.0340016	10.54486296	4.5
3	736.6535369	801.914874	767.4341882	20.17367267	4.2
4	713.197886	735.5509396	725.4096418	6.720954599	1.7
**5**	**716.8215497**	**731.8087794**	**722.8344424**	**4.782562185**	**1.4**
F8	Min	Max	Mea	Std	Friedman rank
1	806.967983	832.0459216	816.2353258	6.393977444	2.5
2	818.4762189	839.132703	832.0035994	6.246104713	4.8
3	814.4292554	837.9631938	826.130897	8.112131024	3.7
4	804.9266199	831.6392397	815.9005992	8.669605501	2
**5**	**807.9658893**	**829.2153031**	**816.0170901**	**6.944601934**	**2**
F9	Min	Max	Mea	Std	Friedman rank
1	946.4796752	1246.362649	1098.183882	85.98095394	3.6
2	1156.251969	1863.438828	1366.668915	193.404622	4.8
3	962.6981116	1446.502576	1130.548742	148.5163763	3.6
4	900	900.454324	900.0543852	0.143313213	1.4
**5**	**900**	**900.454324**	**900.14525**	**0.215063436**	**1.6**
F10	Min	Max	Mea	Std	Friedman rank
1	1253.201646	1853.110618	1660.092579	188.8047919	1.8
2	1865.56741	2576.272945	2226.247908	280.094693	3.9
3	1606.379894	2842.933106	2433.039247	383.3509471	4.3
4	1219.58642	2053.689197	1671.033357	256.6872011	1.8
**5**	**1428.866281**	**2470.065227**	**2023.278799**	**371.3544838**	**3.2**
F11	Min	Max	Mea	Std	Friedman rank
1	1121.056616	1351.061621	1170.322298	66.50450294	3.6
2	1155.610476	1316.113621	1216.945194	49.66220743	4.8
3	1112.767767	1376.628967	1172.268344	83.91536093	3.6
4	1100	1107.959662	1103.164498	3.063114799	1.55
**5**	**1100**	**1108.465748**	**1103.446362**	**2.579264943**	**1.45**
F12	Min	Max	Mea	Std	Friedman rank
1	34957.44717	1824515.957	1070521.002	665396.9586	3.4
2	103930.6099	3345680.463	2159777.369	952833.602	4.4
3	13063.75784	5979329.961	1779387.902	2148543.76	3.5
4	3666.344137	1256058.71	248629.3781	379546.4291	2
**5**	**6607.470699**	**1355186.727**	**213948.7565**	**416742.7894**	**1.7**
F13	Min	Max	Mea	Std	Friedman rank
1	6489.317996	27511.98608	14173.02225	6899.604137	4.1
2	6831.340901	12253.80743	8548.940482	1826.587335	3
3	3777.505241	19687.02187	11837.12948	5591.555417	3.8
4	1830.392928	21222.43017	5354.217666	5975.907637	1.9
**5**	**1703.23811**	**29536.54016**	**8660.477382**	**11111.01566**	**2.2**
F14	Min	Max	Mea	Std	Friedman rank
1	1458.817344	8745.269656	4206.810007	2124.465056	4
2	1528.005129	14508.78978	5625.546454	4525.315463	4.1
3	2272.485198	5978.119543	4096.162229	1303.490734	3.9
4	1426.574873	1442.002045	1433.54484	5.193921871	1.5
**5**	**1424.043186**	**1441.648415**	**1432.097727**	**6.217721714**	**1.5**
F15	Min	Max	Mea	Std	Friedman rank
1	1649.292078	7076.842659	4262.260079	2004.791502	3.5
2	4378.710171	21922.49018	15416.13427	5477.664593	4.8
3	4132.244097	13375.59011	7027.096852	3245.255616	3.7
4	1504.131817	1529.001752	1512.983853	7.837796727	1.4
**5**	**1504.985702**	**1545.639016**	**1517.128402**	**12.45976034**	**1.6**
F16	Min	Max	Mea	Std	Friedman rank
1	1735.421635	1994.158888	1886.832328	86.44952943	3.6
2	1868.908206	2185.781568	2018.337498	116.0386239	4.5
3	1770.664284	2032.921409	1919.903777	90.43678099	3.9
4	1601.252786	1749.89508	1628.51303	45.82796546	1.4
**5**	**1601.649978**	**1644.062079**	**1614.57295**	**11.69988358**	**1.6**
F17	Min	Max	Mea	Std	Friedman rank
1	1737.867541	1764.930099	1754.94338	7.571579343	2.8
2	1757.906259	1843.220082	1777.408016	24.9100037	4.5
3	1753.867683	1841.466687	1780.068551	28.58132935	4.2
4	1727.779812	1769.701725	1745.240276	12.41173641	1.6
**5**	**1726.93527**	**1769.805936**	**1744.499634**	**14.77569089**	**1.9**
F18	Min	Max	Mea	Std	Friedman rank
1	2076.858634	22364.25303	9885.864006	6200.172993	2.9
2	10802.19954	18346.3907	14622.21052	2419.785631	4.1
3	3024.850761	14597.87473	7415.957874	4422.310767	2.2
4	5254.151729	34911.05334	11945.61114	11049.49374	2.6
**5**	**3805.601235**	**41859.54728**	**14466.5231**	**12314.86385**	**3.2**
F19	Min	Max	Mea	Std	Friedman rank
1	1941.885641	12781.20193	7731.901925	3719.181377	4.2
2	2397.337357	3004.026784	2658.436419	215.3843564	3.5
3	1961.792191	138325.3517	36364.07579	51340.57586	4.3
4	1905.613737	1918.214378	1911.297281	3.878175052	1.6
**5**	**1906.422524**	**1934.102187**	**1912.857196**	**8.426329797**	**1.4**
F20	Min	Max	Mea	Std	Friedman rank
1	2022.704493	2176.472805	2083.121805	45.56618073	3.3
2	2081.230426	2214.804498	2151.99144	52.55500153	4.6
3	2055.914971	2159.126457	2097.218649	38.46768192	4.1
4	2000.312173	2020.994959	2004.611417	8.40798481	1.15
**5**	**2000.312173**	**2024.976886**	**2017.287352**	**9.098643559**	**1.85**
F21	Min	Max	Mea	Std	Friedman rank
1	2204.294431	2358.553414	2284.230473	67.31997559	2.7
2	2208.507502	2367.069025	2320.529097	54.77109099	3.8
3	2324.288575	2382.757479	2345.773806	17.97087718	4.2
4	2202.196166	2327.902207	2283.872306	56.35357109	2.1
**5**	**2309.586963**	**2322.422831**	**2314.375189**	**3.482857166**	**2.2**
F22	Min	Max	Mea	Std	Friedman rank
1	2312.045753	2452.600719	2351.731274	53.67288421	4.1
2	2305.046456	2533.346294	2416.806987	69.11582186	4.4
3	2306.762431	2403.5622	2329.641616	34.28069688	3.4
4	2300.344439	2308.210778	2303.101123	2.410145197	1.7
**5**	**2301.210643**	**2306.350614**	**2303.782197**	**1.955927516**	**1.4**
F23	Min	Max	Mea	Std	Friedman rank
1	2637.447728	2717.981524	2672.204755	24.88223237	3.8
2	2642.284071	2718.562066	2683.993832	25.63552155	4.3
3	2626.066179	2743.599284	2675.955657	29.02670441	3.9
4	2609.764217	2647.451379	2617.364803	10.91393414	1.4
**5**	**2611.861738**	**2621.221441**	**2616.909175**	**3.053347342**	**1.6**
F24	Min	Max	Mea	Std	Friedman rank
1	2549.089363	2884.550261	2742.620328	117.851719	3.7
2	2520.717846	2823.430111	2693.1907	125.816325	2.8
3	2519.670451	2838.958925	2755.381171	87.89396049	3.4
4	2500	2768.316463	2730.596862	81.63359036	2.8
**5**	**2500**	**2759.601272**	**2698.260735**	**104.6856615**	**2.3**
F25	Min	Max	Mea	Std	Friedman rank
1	2929.512332	3038.269291	2963.374693	38.8283153	3.9
2	2897.98786	2972.549482	2924.374458	23.84318349	2.3
3	2931.841538	3069.95598	2973.548411	40.38736781	4.5
4	2897.78573	2945.961137	2930.85767	22.0253727	2.4
**5**	**2897.762447**	**2943.611415**	**2920.740965**	**23.98466475**	**1.9**
F26	Min	Max	Mea	Std	Friedman rank
1	2869.821098	3732.387795	3311.92128	288.1035138	3.7
2	3145.011465	3498.225635	3346.917361	135.887357	3.9
3	2972.361824	4124.715064	3474.789206	464.672894	3.8
4	2800	3871.351484	2996.712865	314.3165906	1.65
**5**	**2900**	**3048.626277**	**2964.846001**	**51.63292906**	**1.95**
F27	Min	Max	Mea	Std	Friedman rank
1	3110.468489	3235.062729	3191.927358	38.97006482	4.3
2	3100.023307	3178.344818	3124.354973	22.74906712	3.1
3	3126.512789	3233.874923	3189.645169	31.21676218	4.4
4	3089.308077	3139.5379	3096.378378	15.44103258	1.9
**5**	**3086.889208**	**3095.742537**	**3091.01721**	**3.033904717**	**1.3**
F28	Min	Max	Mea	Std	Friedman rank
1	3100.270563	3718.266624	3395.979997	199.0744766	3
2	3449.81686	3521.949609	3473.176735	20.88370759	4
3	3163.212778	3754.475171	3457.466146	201.9129708	3.4
4	3100	3731.812926	3408.003849	149.2339865	2.5
**5**	**3196.568116**	**3411.821808**	**3383.766947**	**66.76990701**	**2.1**
F29	Min	Max	Mea	Std	Friedman rank
1	3177.728213	3407.71245	3258.845347	68.54650087	3.3
2	3223.859415	3362.32843	3320.19169	47.4061423	4.3
3	3231.97607	3367.577902	3313.198586	44.94383581	4.2
4	3137.190144	3205.923406	3156.023831	19.37161251	1.2
**5**	**3141.129471**	**3276.588712**	**3172.826203**	**40.10175036**	**2**
F30	Min	Max	Mea	Std	Friedman rank
1	45852.59593	7054708.048	2943003.11	2889618.788	3.9
2	79074.40476	3336488.755	532797.2983	1012170.375	3.1
3	157369.3702	19115388.14	3658397.123	6009271.347	3.9
4	4606.133975	1251762.743	303544.4899	472087.4093	2.2
**5**	**6526.421462**	**821874.7304**	**172283.0283**	**342040.0326**	**1.9**

**Table 3:** The p-values obtained by Wilcoxon signed-rank test of ablation experiments.

Function	vs ZOA		vs OZOA		vs PZOA		vs SZOA	
	p-values	h	p-values	h	p-values	h	p-values	h
F1	1.8267e-04	1	1.8267e-04	1	1.8267e-04	1	2.58E-06	1
F3	1.0997e-04	1	1.0997e-04	1	1.0997e-04	1	4.28E-07	1
F4	1.8267e-04	1	1.8267e-04	1	1.8267e-04	1	3.63E-06	1
F5	0.0028	1	3.2984e-04	1	4.3964e-04	1	0.0001	1
F6	1.8267e-04	1	1.8267e-04	1	1.8267e-04	1	1.40E-06	1
F7	0.001	1	1.8267e-04	1	1.8267e-04	1	1.99E-06	1
F8	0.5205	0	7.6854e-04	1	0.0073	1	8.29E-06	1
F9	1.8267e-04	1	1.8267e-04	1	1.8267e-04	1	5.58E-07	1
F10	0.0312	1	0.2123	0	0.0312	1	0.0002	1
F11	1.8267e-04	1	1.8267e-04	1	1.8267e-04	1	7.35E-07	1
F12	0.0036	1	0.0006	1	0.0073	1	0.0004	1
F13	0.064	0	0.1405	0	0.0757	0	0.0051	0
F14	1.8267e-04	1	1.8267e-04	1	1.8267e-04	1	4.71E-06	1
F15	1.8267e-04	1	1.8267e-04	1	1.8267e-04	1	7.45E-07	1
F16	1.8267e-04	1	1.8267e-04	1	1.8267e-04	1	2.14E-06	1
F17	0.1041	0	0.001	1	0.0036	1	1.50E-05	1
F18	0.6232	0	0.1859	0	0.1405	0	0.0832	0
F19	1.8267e-04	1	1.8267e-04	1	1.8267e-04	1	2.31E-06	1
F20	3.2984e-04	1	1.8267e-04	1	1.8267e-04	1	5.63E-07	1
F21	0.5708	0	0.0257	1	1.8267e-04	1	0.0059	0
F22	1.8267e-04	1	4.3964e-04	1	0.002	1	4.21E-06	1
F23	1.8267e-04	1	1.8267e-04	1	1.8267e-04	1	3.63E-06	1
F24	0.0889	0	0.4725	0	0.0211	1	0.2998	0
F25	0.0091	1	0.8501	0	7.6502e-04	1	0.0004	1
F26	0.0257	1	1.8267e-04	1	0.0028	1	0.0006	1
F27	1.8267e-04	1	1.8267e-04	1	1.8267e-04	1	3.00E-06	1
F28	0.3010	0	1.4939e-04	1	0.2666	0	0.0619	0
F29	0.0022	1	3.2984e-04	1	3.2984e-04	1	5.28E-06	1
F30	0.0028	1	0.0113	1	0.0017	1	0.0076	0

C **Analysis of convergence capability**

To analyze the convergence behavior of the traditional Zebra Optimization Algorithm and solution quality at different improvement stages, Fig 3 presents convergence curves comparing algorithm performance. Fig 4 further displays the box plot distributions across all 30 test functions. The convergence analysis reveals that OP-ZOA exhibits minimal initial convergence delay when solving F10, F13, F18, F21, F28, and F30, while demonstrating accelerated convergence during mid-to-late iterations and ultimately achieving superior optimization results.

Fig 4 demonstrates that OP-ZOA consistently generates higher-quality solutions with reduced solution variance compared to competing algorithms, indicating stable performance through its centralized data distribution pattern. However, the algorithm shows susceptibility to local optima in test functions F10, F13, F18, and F25, as evidenced by data points exceeding the central distribution boundaries.

D **Statistical tests**

Statistical evaluation in Table 2 demonstrates that OP-ZOA achieves top-two Friedman rankings for 28 of the 30 test functions, with exceptions occurring only in F10 and F18. The algorithm exhibits susceptibility to local optima in functions F10, F13, and F18, which slightly reduces its robustness. Nevertheless, OP-ZOA maintains top-tier performance in standard deviation rankings across all other test functions, confirming its overall optimization stability. Table 3 presents Wilcoxon signed-rank test results at α=0.05, where most p-values below this threshold support alternative hypothesis $H_{1}$, statistically confirming OP-ZOA’s superiority.

Collectively, the convergence patterns in Figs 3 and 4 and statistical evidence in Tables 2 and 3 validate that the enhanced OP-ZOA algorithm demonstrates significant robustness when evaluated against CEC2017 benchmark functions.

### Simulation comparison and performance test

A **Experimental design**

To verify the performance of the improved OP-ZOA algorithm, it is compared with seven other algorithms: the Bloodsucking Leech Algorithm (BSLO) [43], Spider Wasp Optimization Algorithm (SWO) [44], Parrot Optimization Algorithm (PO) [45], Polar Lights Algorithm (PLO) [46], Red-tailed Hawk Optimization Algorithm (RTH) [47], Bitterling Fish Optimization Algorithm (BFO) [48], and Zebra Optimization Algorithm (ZOA) [34]. The CEC2017 test function consists of a total of 30 objective test functions with search intervals between [−100,100].

B **Analysis of convergence capability**

To comprehensively analyze algorithm convergence and solution quality, we employ convergence plots, fitness trajectories, and box plots. The OP-ZOA’s convergence behavior is evaluated through six metrics: search history, convergence curves, average fitness, population diversity, first-dimension trajectories, and exploration-exploitation ratios.

**Fig 5:**
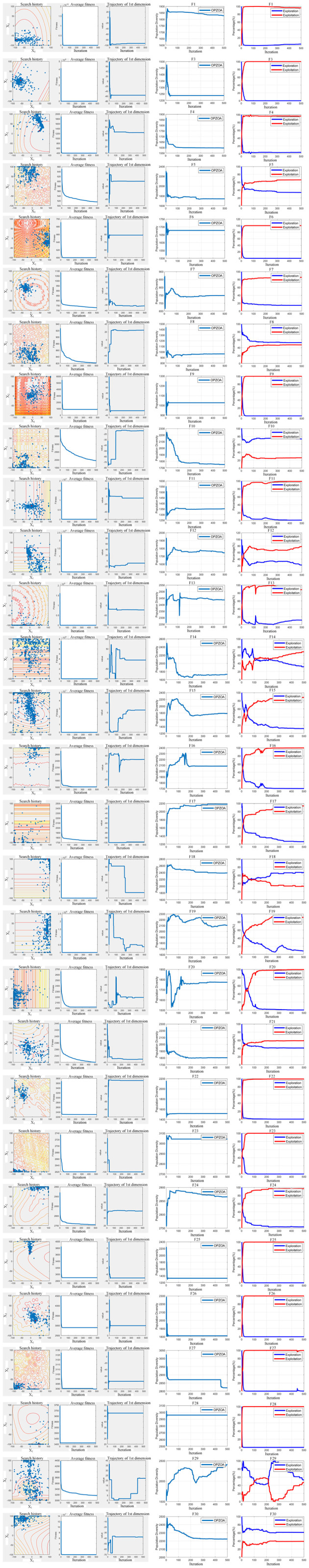
Convergence analysis plot for OP-ZOA (Search history, Average fitness, Trajectory of 1st dimension, Population diversity, Changes in the percentage of exploration and exploitation).

Fig 5 illustrates these analyses, where the first column’s search history reveals individuals initially exploring promising regions before converging toward global optima, demonstrating effective exploration-exploitation balance. Notably, for function F15, OP-ZOA exhibits strong exploratory behavior by extensively searching the upper-left solution space before locating optima in the right region, highlighting its ability to maintain population diversity and avoid local optima. The second column’s average fitness curves demonstrate OP-ZOA’s rapid convergence. While exhibiting strong exploitation for unimodal problems through inter-individual learning, the algorithm occasionally encounters local optima in multimodal and hybrid problems, yet achieves high precision through dynamic elite-pooling guidance. Columns three and four present first-dimension trajectories and population diversity, showing significant initial variation that promotes exploration of high-quality solutions. The fifth column’s exploration-exploitation analysis reveals OP-ZOA’s adaptive balance: rapid exploitation intensification for unimodal functions versus gradual exploration reduction in multimodal scenarios.

**Fig 6:**
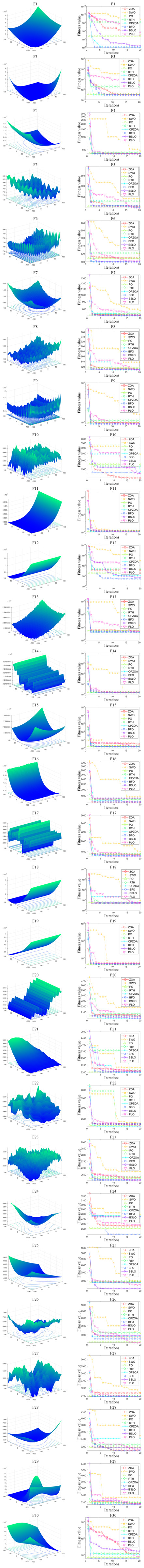
CEC2017 test function images and iteration curves of different algorithms.

**Fig 7:**
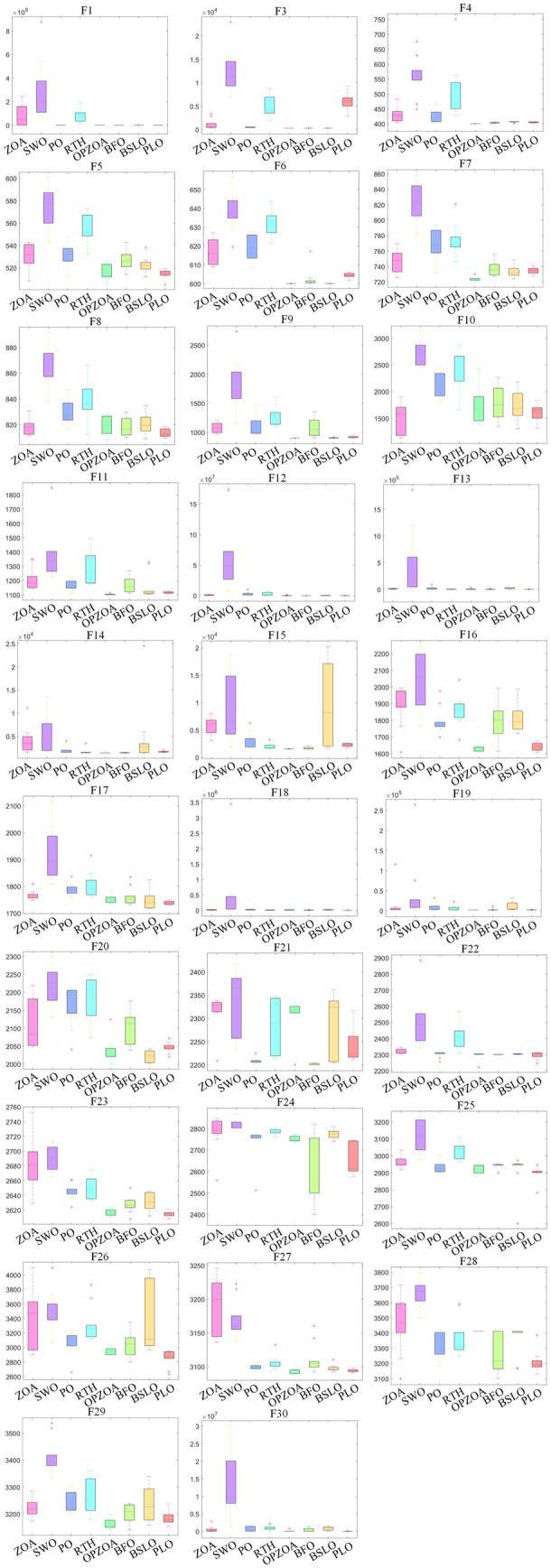
Box plots of different algorithms on CEC2017 test function.

For standardized comparison, all algorithms use identical parameters: population size 30, 10-dimensional search space, and 500 maximum iterations. Fig 6 displays convergence curves from 10 independent runs across all 30 functions, while Fig 7 presents their corresponding box plot distributions.

The convergence analysis in Fig 6 demonstrates that while ZOA shows marginally better convergence performance than SWO, PO, RTH, BFO, BSLO, and PLO algorithms, it still exhibits several limitations including curve flattening, search stagnation, reduced optimization accuracy, and susceptibility to local optima. In contrast, the enhanced OP-ZOA algorithm achieves notable improvements in both convergence speed and solution accuracy compared to the original ZOA, while effectively overcoming local optima through its opposition-based learning and dynamic elite-pooling mechanisms.

Comparative evaluation reveals that OP-ZOA maintains similar initial convergence rates to ZOA while achieving superior final accuracy, owing to its real-time information synchronization and dynamic elite-pooling strategies. Although OP-ZOA shows slightly slower convergence when test functions F8, F10, F18, F20, F21, F28, and F30 - potentially due to exploration-exploitation imbalances – it demonstrates consistently higher accuracy in other test functions. These results confirm that the implemented opposition-based learning and dynamic elite-pooling strategies significantly enhance ZOA’s global optimization capability while reducing its tendency for premature convergence.

Fig 7 demonstrates that OP-ZOA maintains concentrated data distributions and stable performance across multiple runs. However, the OP-ZOA algorithm may fall into local optima in test functions F5, F8, and F10, resulting in a large range of data distribution.

C **Statistical tests**

To rigorously evaluate OP-ZOA’s optimization accuracy, we compared eight algorithms (ZOA, SWO, PO, RTH, OP-ZOA, BFO, BSLO, and PLO) on 30 test functions (dim = 10), conducting 10 independent runs per algorithm. Performance was assessed using seven metrics: minimum value (Min), mean value (Mean), standard deviation (Std), maximum value (Max), Wilcoxon rank-sum test, Wilcoxon signed-rank test, and Friedman test, with detailed results presented in Table 4. The table entries 1–8 correspond to ZOA, SWO, PO, RTH, OP-ZOA, BFO, BSLO, and PLO respectively.

**Table 4:** CEC2017 test function test results.

F1	Min	Max	Mea	Std	Friedman rank
1	16575.2152	2227327272	838538226.3	783134931	6.3
2	1408492784	8951762767	3533114666	2222405803	7.9
3	335572.8265	6636877.162	2175246.835	2491300.689	4.7
4	85790849.47	6290892839	1269862964	1893139246	6.5
**5**	**281.8557859**	**6191.106748**	**2871.955867**	**2234.759932**	**1.7**
6	183.4555062	7034.256651	2863.732887	2619.507074	1.9
7	131.3185511	12740.80249	7339.909114	4551.894298	2.4
8	289242.1413	1862003.699	1052012.593	478637.9453	4.6
F3	Min	Max	Mea	Std	Friedman rank
1	330.0045563	3332.369015	1111.869923	1093.497381	4.7
2	7008.319454	22987.22338	12140.82857	4759.098411	8
3	349.4843137	605.7915646	486.8089534	90.63506483	4.3
4	1925.387728	8654.435908	4735.248551	2262.25907	6.4
**5**	**300**	**300**	**300**	**3.78956E-14**	**1.1**
6	300	300	300	1.13687E-13	1.9
7	300.0385458	307.256834	301.7966475	2.653425641	3
8	2851.293578	9206.720206	5935.412301	1865.970682	6.6
F4	Min	Max	Mea	Std	Friedman rank
1	403.7666471	481.9294632	428.6667056	23.31552792	5.6
2	448.8727864	676.2300379	557.4901227	66.8180372	7.7
3	406.5514085	466.0467682	421.8229631	24.26639873	5.3
4	426.8214188	750.8104533	510.5975079	95.52525821	7
**5**	**400.0894331**	**400.3124602**	**400.1405695**	**0.065217941**	**1**
6	400.9260243	405.7606522	403.3546962	1.538115939	2.4
7	401.2828669	407.2570449	405.8809822	2.092239508	3.9
8	402.69747	406.6955203	404.735793	1.522205778	3.1
F5	Min	Max	Mea	Std	Friedman rank
1	522.4720057	550.5801422	531.1860187	8.491676755	4.9
2	570.8175738	593.629826	580.4524347	8.022244948	7.9
3	522.2943504	561.1153631	539.0849338	13.26613223	5.5
4	536.4808695	576.0800674	558.5280269	14.62024136	6.8
**5**	**507.471481**	**518.6182031**	**514.6771304**	**3.461864366**	**1.9**
6	507.9596674	537.8082977	523.377141	11.07816049	3.6
7	509.9495855	531.7951712	522.3556935	7.430618955	3.3
8	509.7423921	518.4901805	515.6361041	2.642930466	2.1
F6	Min	Max	Mea	Std	Friedman rank
1	608.7385134	627.0101802	616.9982864	7.123983675	5.3
2	619.5769678	656.8542894	638.3183631	10.08774665	7.8
3	608.6032744	627.6242273	619.1193835	6.686656895	5.5
4	621.2398231	643.5225789	632.1295553	7.354412515	7.2
**5**	**600**	**600.0000485**	**600.0000095**	**1.70272E-05**	**1**
6	600.0983123	617.1677244	602.5952178	5.182262222	3.3
7	600.0006643	600.0711492	600.0121035	0.02119391	2
8	601.6486747	606.009465	604.235562	1.389900042	3.9
F7	Min	Max	Mea	Std	Friedman rank
1	725.60906	769.9805289	746.9779793	15.44857019	4.4
2	781.9067613	858.1041662	822.6200754	25.42705833	7.9
3	731.8428481	793.6091359	768.4806113	18.35763213	6.3
4	745.6431043	820.6246781	772.7297362	21.15288818	6.5
**5**	**719.6141275**	**730.2088383**	**723.2357865**	**2.906117205**	**1.2**
6	727.3511179	755.9773281	737.5013473	9.335253724	3.3
7	723.8199131	748.5899086	733.074851	7.525843038	3
8	727.6195097	740.9279197	734.6224851	4.300090289	3.4
F8	Min	Max	Mea	Std	Friedman rank
1	811.5866879	827.0073524	817.0252188	4.958969208	3.3
2	856.8808205	879.2033316	865.2713559	7.317959259	8
3	812.6765656	841.0141782	829.981605	8.316169841	5.7
4	825.8189232	847.5830887	836.2558581	6.812478134	6.4
**5**	**807.892265**	**820.8267994**	**812.1629117**	**4.877453521**	**2**
6	812.9344577	840.7931748	821.8890457	8.899144927	4.4
7	806.9647084	845.8619053	820.5068337	13.41879755	3.5
8	811.0943752	818.8270461	813.9377618	2.307963267	2.7
F9	Min	Max	Mea	Std	Friedman rank
1	970.5610528	1215.111365	1073.475238	85.2320013	5.2
2	1150.553875	2735.316653	1803.259377	437.2457123	7.8
3	960.1874109	1473.229222	1083.006592	163.8416396	5
4	1091.325075	1605.722891	1267.038528	158.7491609	6.5
**5**	**900**	**900**	**900**	**3.78956E-14**	**1**
6	900.908648	1351.96532	1081.209554	145.875915	5.3
7	900.0001816	912.7768522	903.7608335	3.864919485	2.1
8	905.0054192	948.2866037	918.9927616	13.52595142	3.1
F10	Min	Max	Mea	Std	Friedman rank
1	1125.881755	1901.600562	1525.357702	289.880258	2.1
2	2449.04377	3132.008604	2757.801641	231.0242056	8
3	1766.452609	2458.880452	2135.188569	244.6172975	5.7
4	1658.59185	2868.998041	2381.240638	371.1509123	6.4
**5**	**1383.554823**	**2426.034104**	**1744.104147**	**317.7030332**	**3.3**
6	1348.871454	2267.31279	1776.784825	332.4969093	3.7
7	1300.539426	2178.664178	1749.065803	277.6439811	3.8
8	1308.8973	1834.24175	1613.452745	149.7500224	3
F11	Min	Max	Mea	Std	Friedman rank
1	1146.866932	1351.061621	1210.641554	78.19950077	5.4
2	1226.348268	1849.492446	1375.258635	180.544064	7.3
3	1109.554372	1222.711108	1165.222684	38.5654732	4.5
4	1174.60607	1494.706924	1289.859043	115.9916881	6.8
**5**	**1100**	**1113.929391**	**1104.005457**	**3.951451523**	**1.1**
6	1111.340703	1269.159271	1169.444248	54.37784053	4.8
7	1102.9996	1330.306032	1155.219033	89.83841774	3.5
8	1110.213316	1129.05286	1116.981241	6.623290473	2.6
F12	Min	Max	Mea	Std	Friedman rank
1	138113.8863	2678500.175	1000586.783	805614.934	5.2
2	7447399.447	174481235.1	65851179.25	59885329.91	8
3	37498.16737	9976644.553	2854248.855	2938222.566	5.7
4	215013.9097	7343732.217	2666400.226	2559389.433	6.1
**5**	**5539.028735**	**1059458.471**	**212757.4186**	**381364.405**	**2.8**
6	2716.780048	41549.19478	10753.23854	12710.10309	1.2
7	5072.641445	400515.3512	162840.2832	161392.7993	3.6
8	32261.69405	226460.9809	87003.3532	65796.87812	3.4
F13	Min	Max	Mea	Std	Friedman rank
1	7623.774812	41469.04602	19327.47681	11319.9747	6
2	10916.69573	11173394.73	1791834.676	3379647.585	7.9
3	4372.307735	34959.00208	18235.54174	12528.083	5.8
4	1588.469068	20525.73126	6174.122663	7002.031227	3.7
**5**	**1673.448611**	**6813.973241**	**3264.760874**	**1758.362902**	**2.4**
6	1765.127488	24420.09253	7608.717624	7207.151548	3.6
7	1344.659971	30453.26268	10696.43109	10473.98283	4.2
8	1773.683066	4257.467826	2961.954515	720.0697078	2.4
F14	Min	Max	Mea	Std	Friedman rank
1	1458.817344	11105.51472	4051.473263	2867.215267	6.4
2	1597.015722	13582.77851	5383.010238	3975.617147	6.8
3	1536.715976	3932.808663	1966.973823	721.3419476	5.2
4	1456.408353	3463.983706	1701.995852	619.7258854	3.6
**5**	**1424.085006**	**1437.410306**	**1431.780643**	**4.083685933**	**1.1**
6	1424.161694	1522.527913	1479.409702	33.73844561	2.3
7	1439.17472	24468.22197	4863.518554	7016.797814	5.6
8	1547.03467	2084.622107	1682.123868	159.888765	5
F15	Min	Max	Mea	Std	Friedman rank
1	3077.828924	7973.706884	5717.814769	1488.674545	6.7
2	1825.658403	18848.03494	8796.001385	5967.035686	6.7
3	1686.550747	6280.395227	3018.858254	1472.768899	4.9
4	1605.58738	3210.486748	1989.905902	502.4502851	3.5
**5**	**1505.204587**	**1566.165581**	**1516.491727**	**17.71290827**	**1.3**
6	1505.751231	2092.151827	1682.797684	215.0788479	2.1
7	1655.918112	20291.76736	9687.001812	7351.225347	6.5
8	1693.372108	2666.032903	2265.160936	320.3018996	4.3
F16	Min	Max	Mea	Std	Friedman rank
1	1608.935829	1994.106666	1882.953754	119.6136591	5.8
2	1763.55819	2246.869253	2038.472243	179.6103262	7
3	1698.260037	1974.467779	1796.66692	80.69168184	4.5
4	1682.765916	2042.537359	1858.60124	92.71898153	5.9
**5**	**1610.583792**	**1642.044159**	**1623.521293**	**12.43799354**	**1.5**
6	1613.971169	1991.656704	1797.19096	129.7362579	4.5
7	1719.904654	1988.168199	1809.694864	86.173695	4.9
8	1605.891466	1674.319571	1641.383462	22.96611789	1.9
F17	Min	Max	Mea	Std	Friedman rank
1	1727.801088	1812.568164	1763.219648	22.20812283	4.6
2	1783.01561	2058.619377	1902.439119	94.19233875	7.6
3	1751.753517	1792.283371	1770.400301	12.74509563	5.5
4	1745.872452	1946.716636	1829.020846	53.01772456	7.1
**5**	**1719.995739**	**1770.435442**	**1745.991381**	**16.53645744**	**2.5**
6	1740.981768	1815.971862	1758.664374	21.20341148	3.6
7	1705.584503	1827.915175	1745.587302	35.84689786	2.6
8	1732.971498	1757.045329	1745.090264	7.01218969	2.5
F18	Min	Max	Mea	Std	Friedman rank
1	2264.896464	30211.31241	16176.54118	10590.67912	5
2	7241.274721	3447413.864	531224.8558	1041038.756	7.7
3	3107.46883	39762.31364	20600.80986	12360.64881	5.3
4	2891.224257	20215.96433	9529.149395	5852.326974	4
**5**	**2799.447892**	**34819.29553**	**13296.15624**	**12466.85028**	**4.1**
6	1930.245276	19519.09847	6318.159115	5822.402255	2.9
7	3051.184036	36475.65494	16932.9736	13041.29616	4.6
8	2809.891442	5311.111114	3781.398678	763.6734767	2.4
F19	Min	Max	Mea	Std	Friedman rank
1	2457.10942	115850.6172	15518.91567	35323.34845	5.6
2	1970.091137	264527.6274	44018.14141	80335.05771	6.2
3	1990.755554	31616.13202	10027.40749	9811.50048	5.7
4	1986.902342	21960.8408	6100.139621	6319.454235	4.7
**5**	**1905.967394**	**1916.276443**	**1910.050587**	**2.953855312**	**1.1**
6	1902.318979	10895.42057	2988.600347	2795.290651	3.1
7	2238.324619	32100.68782	11903.90983	11269.44617	6.2
8	1979.667515	2696.410484	2193.482536	210.163884	3.4
F20	Min	Max	Mea	Std	Friedman rank
1	2036.51414	2163.07461	2083.682862	45.87762046	4.3
2	2079.719531	2281.952432	2192.563167	67.12236413	7
3	2081.95608	2239.375278	2136.226941	56.90576898	6.4
4	2092.004505	2292.732538	2192.296683	56.97997907	7.4
**5**	**2000.312173**	**2140.711382**	**2029.046561**	**42.10542578**	**1.5**
6	2023.977931	2145.895513	2079.61667	47.29206918	4.1
7	2001.991711	2051.232774	2024.859467	12.98787315	1.9
8	2029.890543	2092.353399	2052.448804	16.69728021	3.4
F21	Min	Max	Mea	Std	Friedman rank
1	2208.366234	2338.948961	2315.801372	38.86621849	6.1
2	2232.344812	2417.219069	2337.98733	66.94467302	6.8
3	2202.765351	2224.084342	2208.865292	5.834646244	2.3
4	2213.479283	2349.348698	2285.290639	59.70901737	5.4
**5**	**2200**	**2334.890322**	**2308.267667**	**38.9186997**	**5**
6	2200	2203.5899	2201.2653	1.643409576	1.2
7	2203.953264	2361.725675	2285.661065	68.91510582	5
8	2211.039702	2316.575109	2244.120663	40.64686163	4.2
F22	Min	Max	Mea	Std	Friedman rank
1	2305.21261	2345.027258	2320.941494	14.83335289	5.4
2	2341.475938	2884.164452	2504.821315	155.4412949	7.6
3	2256.807435	2319.35636	2302.784746	19.61364352	4.3
4	2310.616495	2569.030769	2411.740228	79.788459	7.2
**5**	**2220.64702**	**2306.240511**	**2294.871966**	**26.12100996**	**2.7**
6	2300.3449	2301.646427	2300.880947	0.478977124	1.6
7	2301.482886	2306.891395	2303.870955	1.735629681	3.2
8	2244.982033	2311.097955	2296.21569	23.62335865	4
F23	Min	Max	Mea	Std	Friedman rank
1	2648.857843	2686.559153	2667.937089	13.19923993	6.8
2	2668.407348	2721.668843	2695.599835	17.11828493	7.8
3	2615.515807	2670.705878	2640.440819	16.74461521	4.6
4	2635.992342	2693.242143	2657.616286	19.75757523	5.7
**5**	**2611.886128**	**2629.222785**	**2616.908785**	**5.591937892**	**1.9**
6	2611.837909	2633.949634	2623.545904	7.582477196	2.9
7	2624.666826	2645.849062	2631.10165	6.54037504	4.2
8	2612.257481	2624.648732	2617.475825	3.903308955	2.1
F24	Min	Max	Mea	Std	Friedman rank
1	2560.067433	2847.956771	2779.38621	83.15934048	6.2
2	2793.022006	2871.020023	2821.020218	26.01633341	7.5
3	2512.47851	2773.669657	2737.30513	79.30913737	4
4	2761.242296	2801.50486	2785.43559	11.69860532	5.7
**5**	**2739.970326**	**2773.478758**	**2752.085747**	**12.06095357**	**3.1**
6	2400	2820.236905	2563.585009	154.5801615	2.1
7	2740.152188	2809.452868	2773.574273	22.26689037	5.2
8	2577.33473	2746.313541	2646.93908	68.46290248	2.2
F25	Min	Max	Mea	Std	Friedman rank
1	2917.644367	3033.261959	2962.893015	34.86518792	4.8
2	2977.004032	3232.331038	3116.302797	95.13395283	7.6
3	2898.847736	3001.512496	2935.25168	33.64973888	4
4	2964.554149	3107.020631	3033.378028	48.38316009	7.2
**5**	**2897.745671**	**2946.262084**	**2916.853139**	**24.0244321**	**2**
6	2899.584968	2951.74627	2937.288514	20.03146976	3.7
7	2600.235389	2971.861479	2911.287175	110.7915962	4.4
8	2781.845033	2945.094913	2896.356115	42.39917413	2.3
F26	Min	Max	Mea	Std	Friedman rank
1	2900.269036	4103.975971	3414.547232	412.9773683	6.3
2	3061.983502	4101.436795	3491.593555	277.2502546	6.8
3	2658.383635	3192.052181	3068.27827	159.1305523	4.6
4	3114.407347	3865.7987	3327.023857	248.7778887	5.8
**5**	**2900**	**3036.471881**	**2951.34154**	**49.16760757**	**2.2**
6	2800	3348.177247	3045.37361	173.5043498	3.8
7	2967.628572	4072.55928	3345.59443	462.6603168	4.9
8	2629.03081	2947.539568	2849.785428	112.7888263	1.6
F27	Min	Max	Mea	Std	Friedman rank
1	3135.818817	3245.841048	3191.919102	41.22001047	7.6
2	3138.280586	3222.512005	3170.177706	27.22872959	7.3
3	3093.077417	3105.329246	3099.011881	4.176261257	4
4	3096.106137	3132.481898	3105.872331	10.21789795	5.3
**5**	**3089.308077**	**3095.742534**	**3091.449238**	**2.7141825**	**1.5**
6	3092.078591	3160.329732	3111.371741	21.97358853	4.8
7	3092.231903	3110.272728	3097.813243	4.933448022	3.4
8	3090.998521	3097.979498	3093.948514	2.169979703	2.1
F28	Min	Max	Mea	Std	Friedman rank
1	3100.159856	3717.170819	3469.826838	197.9676581	5.9
2	3495.46385	3798.33968	3662.853155	93.10468187	7.8
3	3183.284111	3412.05297	3335.807602	87.30098174	4.1
4	3248.903993	3591.538149	3361.363577	126.0718512	3.8
**5**	**3411.821808**	**3411.821808**	**3411.821808**	**0**	**5.25**
6	3100	3411.821808	3258.200412	132.3450879	2.45
7	3167.144189	3411.821853	3360.468569	101.1325845	4.9
8	3128.704008	3385.253672	3210.484085	69.0347229	1.8
F29	Min	Max	Mea	Std	Friedman rank
1	3173.335658	3284.363349	3223.644607	37.28343529	4.7
2	3332.680514	3537.289258	3408.669336	67.41315314	8
3	3201.251709	3304.946563	3244.835778	40.82601204	5.4
4	3179.869002	3359.481894	3263.888136	63.20522625	5.5
**5**	**3139.583133**	**3197.70831**	**3162.401734**	**19.07604531**	**1.5**
6	3139.781543	3238.944118	3203.776409	35.02941524	3.5
7	3158.4881	3339.832788	3237.197221	65.5529966	4.5
8	3152.721234	3236.870012	3182.017487	24.5434393	2.9
F30	Min	Max	Mea	Std	Friedman rank
1	13801.47672	2901237.856	631478.3363	872171.6138	4.5
2	1200812.433	30127845.18	12916297.56	9971114.913	7.7
3	5005.371321	1806356.076	737620.5276	719188.5257	4.6
4	14486.28797	2192222.87	1020844.673	681473.8599	5.5
**5**	**11782.95394**	**821874.7304**	**182447.194**	**336740.8715**	**3.2**
6	3511.090558	1363710.336	433413.3449	567302.7518	3.4
7	27144.44118	1570322.481	828241.8122	534344.5567	5.2
8	5302.869432	88254.81359	23639.04015	26061.41858	1.9

Table 4 demonstrates that while OP-ZOA may experience exploration-exploitation imbalances in functions F10, F18, F21, F24, and F28 - reflected in its suboptimal Friedman rankings for these cases – it achieves top-two performance rankings in the remaining 25 test functions, indicating statistically significant differences between algorithm groups. Across the 30 test functions, OP-ZOA shows susceptibility to local optima in F2, F10, F18, F20, F21, and F22, as evidenced by reduced standard deviation rankings. However, for all other functions, it maintains top-two standard deviation rankings with consistently smaller values than ZOA, confirming that the implemented opposition-based learning and dynamic elite-pooling strategies successfully enhance both robustness and stability.

Table 5 gives the p-values obtained by the Wilcoxon signed-rank test for OP-ZOA and each comparison algorithms. From the results, it can be concluded that the vast majority of p-values are less than 0.05, i.e., the vast majority of results accept the alternative hypothesis H_1_, which validates the superiority of OP-ZOA.

**Table 5:** The p-values obtained by Wilcoxon signed-rank test.

Function	vs ZOA		vs SWO		vs PO		vs RTH		vs BFO		vs BSLO		vs PLO	
	p-values	H	p-values	H	p-values	H	p-values	H	p-values	H	p-values	H	p-values	H
F1	1.8267e-04	1	1.8267e-04	1	1.8267e-04	1	1.8267e-04	1	0.3447	0	6.20e-11	1	1.8267e-04	1
F3	1.4077e-04	1	1.4077e-04	1	1.4077e-04	1	1.4077e-04	1	0.0028	1	1.4077e-04	1	1.4077e-04	1
F4	1.8267e-04	1	1.8267e-04	1	1.8267e-04	1	1.8267e-04	1	1.8267e-04	1	1.8267e-04	1	1.8267e-04	1
F5	0.0091	1	1.8267e-04	1	0.0036	1	1.8267e-04	1	0.0073	1	0.0640	0	0.6232	0
F6	1.6211e-04	1	1.6211e-04	1	1.6211e-04	1	1.6211e-04	1	1.6211e-04	1	1.6211e-04	1	1.6211e-04	1
F7	3.2984e-04	1	1.8267e-04	1	1.8267e-04	1	1.8267e-04	1	4.3964e-04	1	0.0013	1	3.2984e-04	1
F8	0.7913	0	1.8267e-04	1	0.0376	1	0.0036	1	5.85e-07	1	1.0	0	0.1041	0
F9	8.7450e-05	1	8.7450e-05	1	8.7450e-05	1	8.7450e-05	1	8.7450e-05	1	8.7450e-05	1	8.7450e-05	1
F10	0.2413	0	1.8267e-04	1	0.014	1	0.0022	1	7.09e-08	1	0.7337	0	0.4727	0
F11	1.8267e-04	1	1.8267e-04	1	2.4613e-04	1	1.8267e-04	1	2.4613e-04	1	0.0022	1	7.6854e-04	1
F12	0.0046	1	1.8267e-04	1	0.001	1	0.001	1	0.0113	1	0.2123	0	0.1212	0
F13	0.0211	1	3.2984e-04	1	0.0312	1	0.7913	0	0.3447	0	0.0140	1	2.90e-06	1
F14	1.8267e-04	1	1.8267e-04	1	1.8267e-04	1	1.8267e-04	1	0.0173	1	1.8267e-04	1	1.8267e-04	1
F15	1.8267e-04	1	1.8267e-04	1	1.8267e-04	1	1.8267e-04	1	0.0312	1	1.8267e-04	1	1.8267e-04	1
F16	0.0028	1	1.8267e-04	1	1.8267e-04	1	1.8267e-04	1	0.0028	1	1.8267e-04	1	3.29e-07	1
F17	0.0257	1	1.8267e-04	1	0.001	1	7.6854e-04	1	0.7913	0	9.28e-07	1	0.0211	1
F18	0.6232	0	7.6854e-04	1	0.1212	0	0.8501	0	0.089	0	6.58e-05	1	0.0211	1
F19	1.8267e-04	1	1.8267e-04	1	1.8267e-04	1	1.8267e-04	1	0.0028	1	1.8267e-04	1	1.8267e-04	1
F20	0.0022	1	1.8267e-04	1	5.8284e-04	1	3.2984e-04	1	0.0036	1	0.7913	0	0.0757	0
F21	0.1405	0	0.089	0	0.0028	1	0.9698	0	0.0025	1	6.12e-07	1	0.0140	1
F22	2.4613e-04	1	1.8267e-04	1	0.0211	1	1.8267e-04	1	0.0036	1	0.2123	0	0.089	0
F23	1.8267e-04	1	1.8267e-04	1	1.8267e-04	1	1.8267e-04	1	0.0091	1	0.0046	1	4.68e-10	1
F24	0.0058	1	1.8267e-04	1	0.1405	0	4.3964e-04	1	0.0538	0	1.29e-07	1	0.0022	1
F25	0.0058	1	1.8267e-04	1	0.064	0	1.8267e-04	1	0.0155	1	0.0211	1	3.11e-08	1
F26	0.0139	1	1.8267e-04	1	0.0072	1	1.8267e-04	1	0.1285	0	0.0013	1	0.0139	1
F27	1.8267e-04	1	1.8267e-04	1	0.001	1	1.8267e-04	1	4.3964e-04	1	0.0013	1	0.0312	1
F28	0.1153	0	6.3864e-05	1	0.0172	1	0.0172	1	0.0022	1	3.32e-07	1	6.3864e-05	1
F29	7.6854e-04	1	1.8267e-04	1	1.8267e-04	1	3.2984e-04	1	0.0113	1	0.0028	1	1.76e-07	1
F30	0.0452	1	1.8267e-04	1	0.0257	1	0.0057	1	0.7337	0	0.0022	1	8.38e-06	1

In conclusion, the experimental results presented in Figs 5–7 and Tables 4 and 5 demonstrate that OP-ZOA achieves strong performance across the CEC2017 test functions, validating the effectiveness of the proposed improvement strategies.

D **Sensitivity analysis**

The proposed OP-ZOA algorithm employs two key parameters: the escape coefficient (R) and the escape strategy probability threshold (Ps). A sensitivity analysis was conducted by systematically varying these parameters while maintaining all other parameters constant.

To examine the influence of the escape coefficient (R) and escape strategy probability threshold (Ps) on OP-ZOA’s performance, we performed experiments using the CEC2017 test functions with a dimensionality of 10, population size of 30, and maximum iteration count of 500. Each configuration was independently executed 10 times while maintaining fixed values for all other parameters.

**Fig 8:**
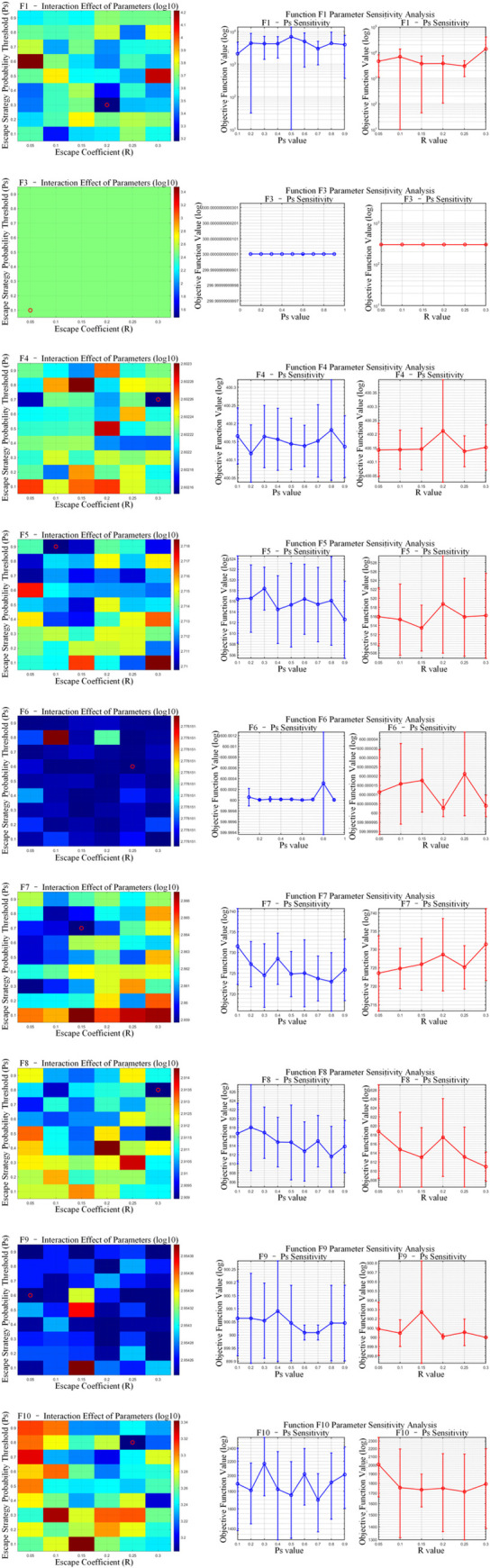
Escape coefficient (R) and escape strategy probability threshold (Ps) unimodal functions analysis.

**Fig 9:**
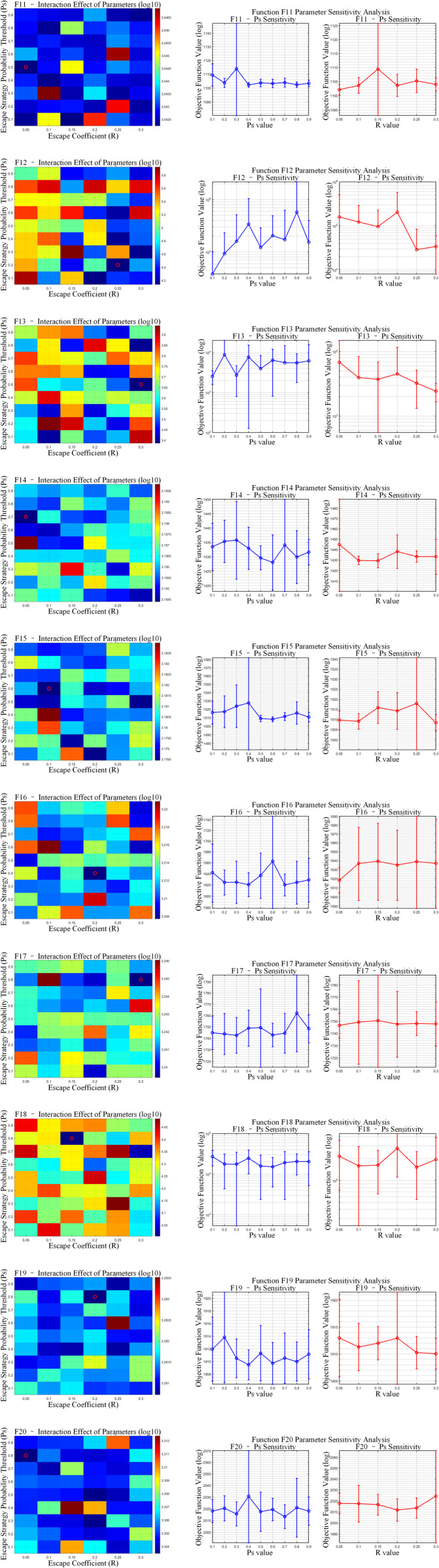
Escape coefficient (R) and escape strategy probability threshold (Ps) hybrid functions analysis.

**Fig 10:**
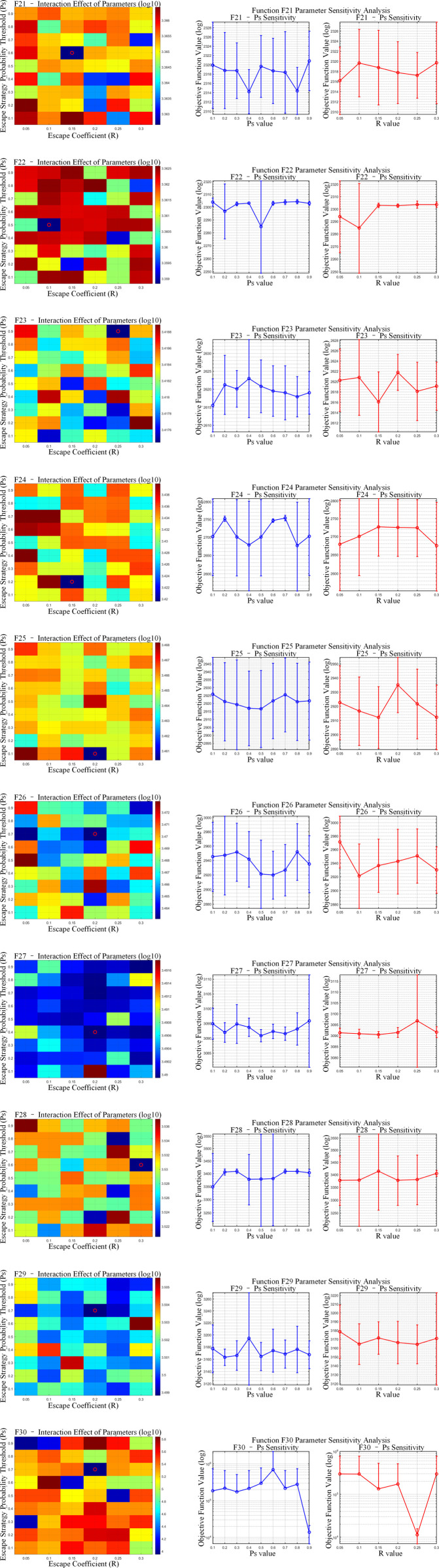
Escape coefficient (R) and escape strategy probability threshold (Ps) composition functions analysis.

**Fig 11:**
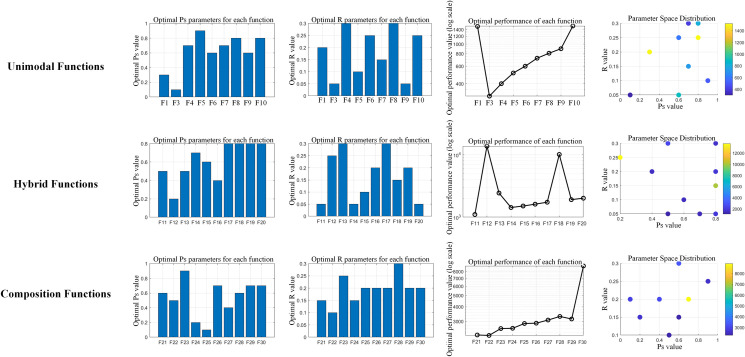
CEC2017 Summary Analysis of Optimal Function Parameters.

The tested R values included 0.05, 0.1, 0.15, 0.2, 0.25, and 0.3, while Ps values ranged from 0.1 to 0.9 in 0.1 increments. Figs 8–10 present sensitivity analyses of R and Ps values across unimodal, hybrid, and composition benchmark functions. The figure presents a dual-component visualization comprising a two-parameter interaction heatmap and a single-parameter sensitivity analysis plot. Within the heatmap, optimal parameter combinations are distinctly marked by red circular indicators. The results (Fig 11 and Table 6) reveal correlation coefficients below 0.1 between R and Ps values across all CEC2017 test functions, indicating that OP-ZOA’s performance remains relatively unaffected by variations in these parameters for unimodal, multimodal, and hybrid function types.

**Table 6:** Sensitivity analysis of escape coefficient (R) and escape strategy probability threshold (Ps) (based on Mean value).

Function	Optimal parameter combination			Correlation coefficient	
	Ps	R	Optimal performance value	Ps	R
F1	0.3	0.2	1.51e + 03	−0.0772	0.0698
F3	0.1	0.05	3.00e + 02	0.0401	0.0076
F4	0.7	0.3	4.00e + 02	−0.0025	0.0043
F5	0.9	0.1	5.13e + 02	−0.0616	−0.0007
F6	0.6	0.25	6.00e + 02	0.0115	0.0883
F7	0.7	0.15	7.23e + 02	0.0444	−0.046
F8	0.8	0.3	8.11e + 02	0.0093	0.0199
F9	0.6	0.05	9.00e + 02	−0.0318	0.0179
F10	0.8	0.25	1.53e + 03	0.0203	−0.0293
F11	0.5	0.05	1.10e + 03	−0.0046	0.0317
F12	0.2	0.25	1.37e + 04	−0.0454	−0.0187
F13	0.5	0.3	2.43e + 03	−0.0123	−0.0022
F14	0.7	0.05	1.43e + 03	−0.0883	0.0223
F15	0.6	0.1	1.51e + 03	−0.0234	−0.0255
F16	0.4	0.2	1.61e + 03	−0.0073	0.0139
F17	0.8	0.3	1.74e + 03	0.0517	−0.0306
F18	0.8	0.15	1.01e + 04	0.0039	0.0075
F19	0.8	0.2	1.91e + 03	0.0399	−0.0993
F20	0.8	0.05	2.01e + 03	−0.0444	−0.0281
F21	0.6	0.15	2.30e + 03	−0.0271	0.0239
F22	0.5	0.1	2.28e + 03	−0.0323	−0.0186
F23	0.9	0.25	2.61e + 03	0.0017	0.0121
F24	0.2	0.15	2.63e + 03	0.0734	−0.0574
F25	0.1	0.2	2.89e + 03	−0.0299	0.018
F26	0.7	0.2	2.90e + 03	−0.051	−0.0267
F27	0.4	0.2	3.09e + 03	0.0019	−0.0072
F28	0.6	0.3	3.32e + 03	0.0164	0.0609
F29	0.7	0.2	3.15e + 03	−0.0525	−0.05
F30	0.7	0.2	8.92e + 03	−0.0316	−0.0249

## Optimization problem application

To evaluate OP-ZOA’s practical effectiveness in engineering applications, we tested it on the local optima avoidance challenge in artificial potential field (APF) path planning, comparing its performance with seven algorithms: SWO, PO, RTH, BFO, BSLO, PLO, and ZOA. The APF optimization task specifically addresses local optima escape to generate optimal collision-free paths with minimal length. We assessed algorithm performance using three critical metrics: fitness value, path length, and computation time. Comparative analysis of convergence behavior, total path lengths, and computational efficiency confirmed OP-ZOA’s superior performance.

**Fig 12:**
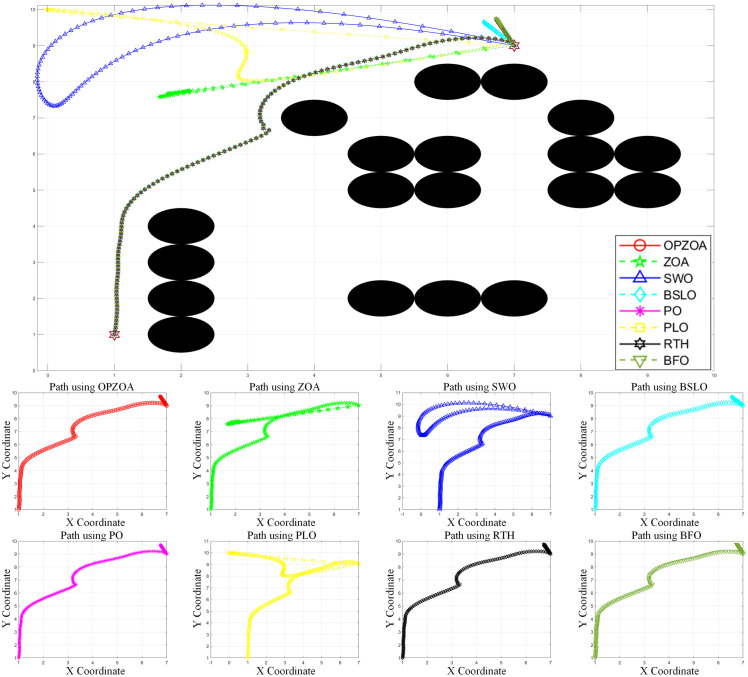
Path planning chart for Environment 1 (10 × 10, destination containing obstacles). Fig 12 demonstrates the path planning performance in a 10 × 10 environment from start point (1,1) to target (7,9). The traditional APF method becomes trapped in a local optimum at (6.97,9.05), exhibiting endpoint oscillations. In contrast, OP-ZOA and the seven comparison algorithms successfully escape this local optimum, achieving collision-free path completion. This comparative result highlights OP-ZOA’s effectiveness in overcoming the characteristic limitations of APF methods.

**Fig 13:**
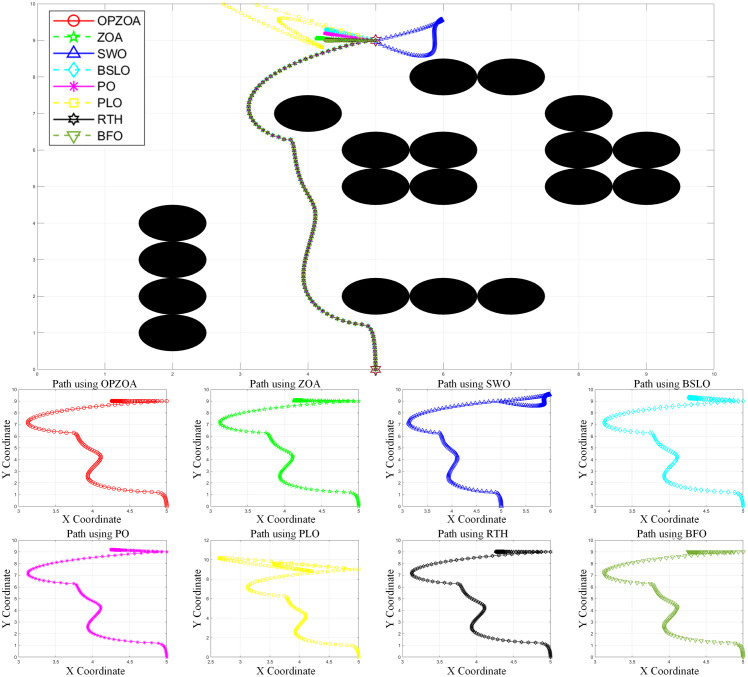
Path planning chart for Environment 2 (10 × 10, destination containing obstacles). Fig 13 illustrates path planning performance in a 10 × 10 environment from start point (5,0) to target (5,9). The traditional APF method becomes trapped in a local optimum at (4.93,9.0), resulting in endpoint oscillations. However, OP-ZOA and all seven comparison algorithms successfully overcome this local optimum, completing collision-free path planning. These results further demonstrate the superior capability of OP-ZOA in handling local optima compared to traditional APF approaches.

**Fig 14:**
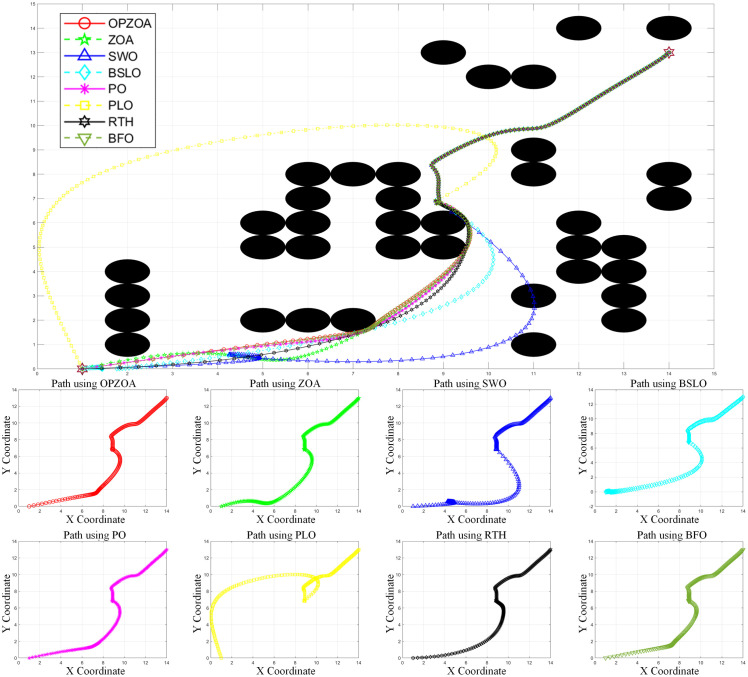
Path planning chart for Environment 3 (15 × 15, U-shaped obstacles). Fig 14 presents path planning results in a 15 × 15 environment from start point (14,13) to target (1,0). The APF method encounters a U-shaped obstacle at (8.79,6.98) and becomes trapped in a local optimum. While OP-ZOA and six other comparison algorithms successfully escape this local optimum and complete collision-free path planning, the SWO algorithm fails due to obstacle collisions. This comparative analysis demonstrates OP-ZOA’s robust performance in complex obstacle environments where traditional methods like APF and certain optimization algorithms (SWO) exhibit limitations.

**Fig 15:**
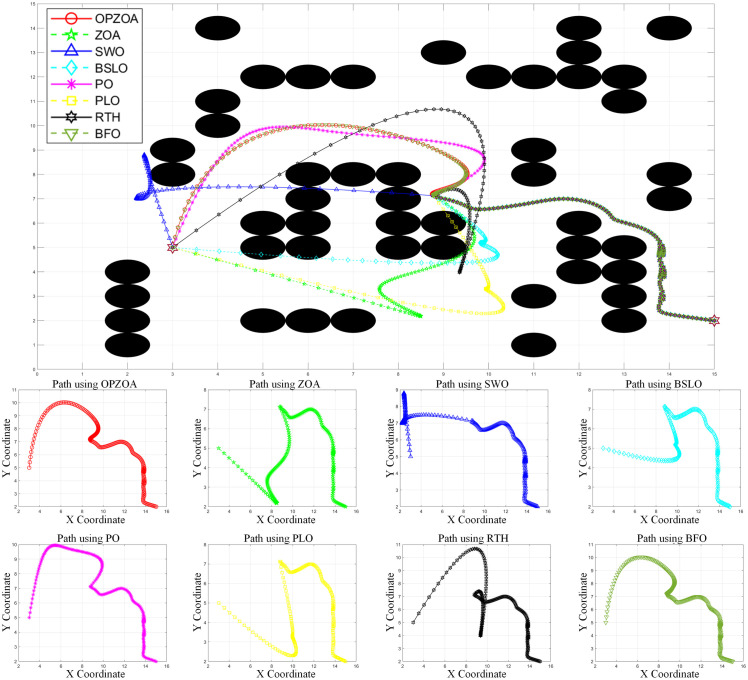
Path planning chart for Environment 4 (15 × 15, U-shaped obstacles). Fig 15 demonstrates path planning performance in a 15 × 15 environment from start point (15,2) to target (3,4). The traditional APF method encounters a U-shaped obstacle at (8.82,6.9) and becomes trapped in a local optimum. While OP-ZOA and five other algorithms successfully escape this local optimum and achieve collision-free path completion, the SWO, BSLO, and PLO algorithms fail due to obstacle collisions. These findings further substantiate OP-ZOA’s enhanced robustness in complex navigation environments where multiple comparative algorithms demonstrate performance constraints.

**Fig 16:**
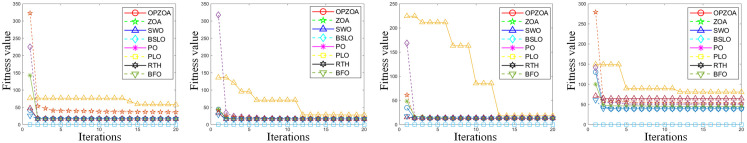
Iteration curves of different algorithms.

In assessing OP-ZOA’s performance for mobile robot navigation, we identified two key scenarios causing APF algorithm entrapment in local optima and consequent target unreachability: U-shaped obstacles and proximal endpoint obstacles. Four specialized environmental configurations were designed to evaluate these local optima scenarios. The experimental outcomes, depicted in Figs 12–15, include: 10 × 10 environment analyses (Figs 12 and 13), 15 × 15 environment evaluations (Figs 14 and 15), eight-algorithm convergence comparisons (Fig 16), post-optimization path length measurements (Table 7), and assessment of different environmental path lengths (Fig 13).

**Table 7:** Path data for different algorithms in four different environments.

Distance	OP-ZOA	ZOA	SWO	BSLO	RTH	BFO	PO	PLO
Environment1	**28.120800m**	47.815022m	64.967193m	28.180988m	28.127857m	28.127778m	28.124672m	66.998669m
Environment2	**26.836157m**	26.967125m	38.162917m	26.959205m	26.836158m	26.836158m	26.910702m	39.324305m
Environment3	**23.206549m**	42.428510m	86.438346m	31.454588m	29.423560m	31.403337m	31.682830m	50.744765m
Environment4	**21.877633m**	22.212336m	27.097780m	22.734171m	22.027204m	21.878510m	21.923889m	30.901953m

**Fig 17:**
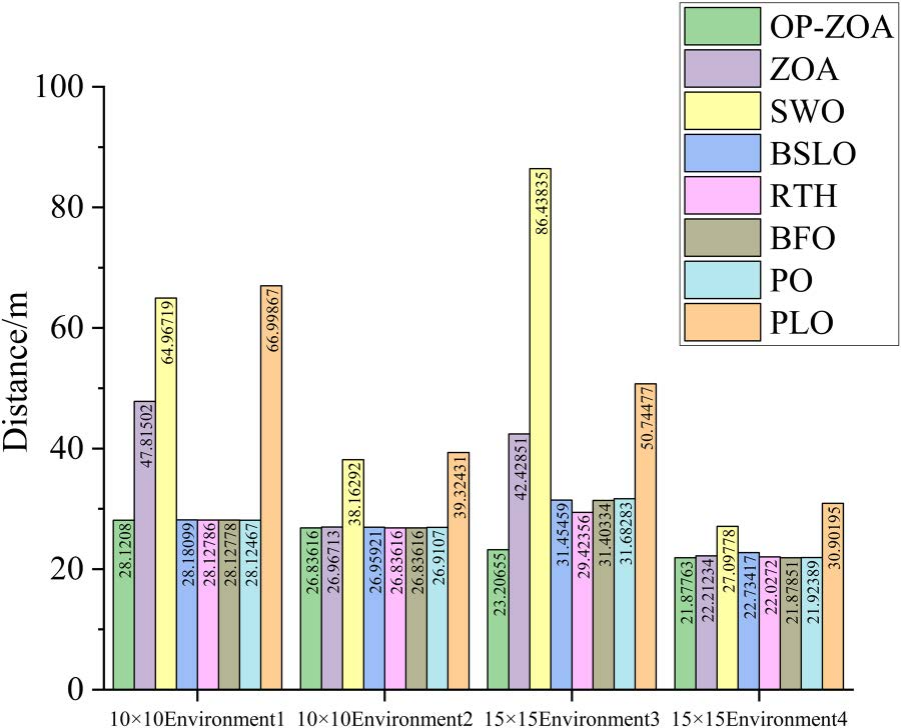
Planning path distances for four different algorithms in four different environments.

Fig 17 and Table 7 demonstrate that OP-ZOA consistently achieves the shortest path lengths after escaping local optima across all experimental settings. In Setting 1, path lengths were reduced by an average of 13.642m (22.316%). Similar improvements were observed in Setting 2 (3.449m, 8.951%), Setting 3 (12.983m, 33.297%, excluding SWO), and Setting 4 (0.133m, 0.6%, excluding SWO, BSLO, and PLO). Overall, OP-ZOA outperformed other algorithms by an average of 16.291% in path length reduction.

Fig 16 further reveals OP-ZOA’s superior convergence rate when handling artificial potential field (APF) challenges, particularly in complex scenarios where optimal solutions are difficult to obtain. These results confirm OP-ZOA’s effectiveness in addressing path planning challenges caused by local minima, while successfully balancing exploration and exploitation. The algorithm demonstrates strong stability and practical engineering value, showing promising potential for solving complex real-world optimization problems.

## Conclusion

In this paper, an enhanced Zebra Optimization Algorithm (OP-ZOA) was proposed. The proposed algorithm integrates Opposition-Based Learning and Dynamic Elite-Pooling strategies. In order to overcome the problem of late search stagnation caused by the lack of population diversity, the good point set-Elite Opposition-Based Learning mechanism is introduced in the initialization of the population. Additionally, a Real-time Information Synchronization mechanism is incorporated into the searcher position update to address the deficiency of the OP-ZOA algorithm in balancing global and local optimization. By introducing a dynamic elite-pooling strategy, three different fitness factors are added, and the fitness factors are randomly selected so as to improve the ability of the algorithm to obtain the global optimal solution, which in turn improves the global optimization accuracy and speed of the OP-ZOA algorithm.

In this paper, the optimization performance of OP-ZOA is verified using CEC2017 test functions. Seven excellent meta-search algorithms proposed in recent years are compared with OP-ZOA on the CEC2017 benchmark function. These algorithms include ZOA, SWO, BSLO, RTH, BFO, PO, and PLO. Then, Wilcoxon signed-rank test and Friedman’s test are performed on the results of the runs of OP-ZOA and its competitors on the CEC2017 benchmark function. The experimental results show that for different types of test functions, the OP-ZOA search algorithm, which contains both opposition-based learning and dynamic elite-pooling strategies, exhibits better convergence speed and accuracy, and is competitive and stable. In addition, a convergence analysis was performed. OP-ZOA was then used to solve the APF artificial potential field method trapped in a locally optimal design problem to validate the ability of OP-ZOA to solve engineering problems. The core results obtained in this study are summarized as follows:

1) OP-ZOA exhibits stronger performance in terms of global optimization and local exploitation compared to the results of competitors’ test function runs at CEC2017.
2) OP-ZOA converges faster on F2 ~ F4, F6, F7, F9, F11, F13, F14, F19, F20, F22, F23, F27, and F29 of the CEC2017 test function compared to its competitors.
3) Wilcoxon signed rank test yields p-values mostly less than 0.05, indicating that OP-ZOA’s results are significantly different from those of its competitors.
4) In Friedman test, the algorithm did not rank first on F10, F12, F18, F21, F22, F24, F28 and F30 and ranked first on the rest of the tested functions.
5) OP-ZOA shows strong scalability on most of the functions in the CEC2017 benchmark set.
6) OP-ZOA algorithm shows some advantages in solving the problem of APF artificial potential field method falling into local optimal design. OP-ZOA algorithm can jump out of the local optimum in four different environments set up in the experiments, and the total paths planned are all the shortest path lengths, and the path lengths are shortened than other optimization algorithms by an average of 7.55175m, which accounts for 16.291%. Among them, in experimental environment 1, the paths are shortened by 13.642m on average, accounting for 22.316%; in experimental environment 2, the paths are shortened by 3.449m on average, accounting for 8.951%; in experimental environment 3 (excluding the SWO algorithm), the paths are shortened by 12.983m on average, accounting for 33.297%; and in experimental environment 4 (excluding the SWO, BSLO and PLO algorithms), the paths are shortened by 0.133m on average, accounting for 33.297%. were shortened by 0.133m on average, accounting for 0.6%.

From the results of this paper, it can be concluded that OP-ZOA has a broad research prospect. However, the introduction of OP-ZOA algorithm inevitably leads to an increase in running time. Therefore, other valuable future research based on this study includes:

1) Providing a multi-objective optimization version of OP-ZOA.
2) Continuously improving on the existing basis in terms of the selection of bootstrapping individuals (e.g., fitness-distance balance), the balance between exploration and exploitation (e.g., parameter or new search operation), and the updating mechanism (e.g., natural survivor method) to ensure the optimization performance while reducing the runtime overhead, and improve computational efficiency.
3) Propose a fusion algorithm with better performance by fusing OP-ZOA and other meta-heuristic algorithms.
4) Integrating the OP-ZOA algorithm into ROS for practical simulation verification of mobile robots.
5) Apply OP-ZOA to different practical optimization problems.
